# Oral Delivery of a Probiotic Induced Changes at the Nasal Mucosa of Seasonal Allergic Rhinitis Subjects after Local Allergen Challenge: A Randomised Clinical Trial

**DOI:** 10.1371/journal.pone.0078650

**Published:** 2013-11-15

**Authors:** Kamal Ivory, Andrew M. Wilson, Prasanna Sankaran, Marta Westwood, Justin McCarville, Claire Brockwell, Allan Clark, Jack R. Dainty, Laurian Zuidmeer-Jongejan, Claudio Nicoletti

**Affiliations:** 1 Gut Health and Food Safety Strategic Programme, Institute of Food Research, Colney, Norwich, United Kingdom; 2 Norwich Medical School, University of East Anglia, Norwich, United Kingdom; 3 Department of Experimental Immunology, Academic Medical Center, Amsterdam, The Netherlands; University of California Los Angeles, United States of America

## Abstract

**Objective:**

To determine effects of probiotic consumption on clinical and immunological parameters of seasonal allergic rhinitis (SAR) in an out-of-season single nasal allergen challenge.

**Methods:**

In a study registered at ClinicalTrials.Gov (NCT01123252), a 16-week dietary intervention was undertaken in 60 patients with allergic rhinitis (>16 years old). Using a double-blinded, placebo-controlled anonymised design, the patients were divided equally into two groups. One group was given a dairy drink containing *Lactobacillus casei* Shirota to ingest daily while the other consumed a similar drink without bacteria. Participants attended the clinic on two consecutive days before the intervention and then again at the end of the study period. On the first day of each 2-day visit, following clinical examination, assessments were made of total nasal symptoms scores and peak nasal inspiratory flow. Nasal scrapings, nasal lavage and blood were collected for laboratory analyses of cellular phenotypes, soluble mediator release and *in vitro* responses to pollen allergen. These procedures were repeated 24 hours following nasal allergen challenge.

**Results:**

Prior to and following intervention there were no detectable differences between study groups in measured clinical outcome. After intervention, there were differences between groups in their percentages of CD86+ epithelial cells (*p* = 0.0148), CD86+CD252+ non-epithelial cells (*p* = 0.0347), sIL-1RII release (*p* = 0.0289) and IL-1β (*p* = 0.0224) levels at the nasal mucosa. Delivery of probiotic also suppressed production of sCD23 (*p* = 0.0081), TGF-β (*p* = 0.0283) and induced increased production of IFN-γ (*p* = 0.0351) in supernatants of cultured peripheral blood.

**Conclusions & Clinical Relevance:**

This study did not show significant probiotic-associated changes with respect to the primary clinical endpoint. An absence of overt clinical benefit may be due to an inability of single nasal challenges to accurately represent natural allergen exposure. Nevertheless, oral delivery of probiotics produced changes of the immunological microenvironment at the nasal mucosa in individuals affected by SAR.

**Trial Registration:**

ClinicalTrials.Gov NCT01123252

## Introduction

Epidemiological studies and recent experimental research support the idea that microbial stimulation of the immune system can influence development of tolerance to innocuous allergens [1]. This makes the gastrointestinal microbiota composition of particular interest, as it provides a major source of immune stimulation and that seems to be a prerequisite for the development of oral tolerance. In allergic diseases, there is thought to be a dysregulation in the expression of T helper 1 (Th1) and T helper 2 cytokines (Th2) which can be alleviated to some extent by *Lactobacilli* belonging to the natural intestinal microflora. One of the proposed mechanisms for this includes direct interaction of *Lactobacilli* with epithelial cells to modify their production of inflammatory mediators [2].In a study of patients with allergic rhinitis out of the pollen season, 4 weeks' treatment with *L. casei* reduced the number of CD16/CD56 cells, *L. plantarum* decreased production of IL-5 and IL-13 and both probiotics reduced the amount of birch-pollen specific IgE [3]. However, in this study immune parameters were determined in serum and peripheral blood mononuclear cell cultures and the patients were not exposed to allergen, which makes it impossible to evaluate the impact of these findings on allergic disease. In our previous study involving patients with allergic rhinitis receiving *L casei* Shirota (LcS) or placebo for 5 months, we found a significant reduction in levels of antigen-induced IL-5, IL-6 and IFN-gamma production during the pollen season in the probiotic compared with placebo supplemented group. Meanwhile, serum levels of specific IgG increased and IgE decreased in the probiotic group [4].

Several trials have investigated the clinical benefit of administering probiotic drinks to patients with seasonal allergic rhinitis [5] (SAR). The available data are inconclusive and often contradictory [6], [7], [8]. We have therefore conducted a double-blinded, placebo-controlled study to test our hypothesis that daily consumption of *Lc*S over a period of four months will result in clinical improvement in an allergen challenge model of allergic rhinitis, with corresponding immunological changes at the nasal mucosa and in blood. There are specific difficulties associated with clinical trials in allergic rhinitis, such as variations between subjects in the extent of pollen sensitization due to differences in exposure to pollen. In order to control such variability we have used a single-dose allergen challenge in the clinic. Total Nasal Symptom Score (TNSS) was selected as the primary end-point because the nasal symptom questionnaire is a convenient, reliable and valid method for assessing nasal symptom severity. Its principal use was to act as a primary outcome measure by comparing scores before and after treatment. Secondary end-points included peak nasal inspiratory flow (PNIF) as a simple, objective measurement of nasal airflow and laboratory tests for local and systemic parameters of immunity.

## Materials and Methods

CONSORT checklist and the protocol for this trial are available as supporting information; see Checklist S1 and Protocol S1.

### Ethics

The study protocol was approved by the local Institute of Food Research Human Research Governance Committee (HRGC ref:IFR05/2009) and national Hertfordshire Research Ethics Committee NREC (ref:09/H0311/77). All subjects gave written, informed consent before participating in the study.

### Probiotic and placebo drinks

All drinks were supplied by Yakult Europe B.V. with HACCP certification for the safety of materials, production process and microbiological analysis (issued by Nederlandse Organisatie voor Toegepast Natuurwetenschappelijk Onderzoek, the Netherlands). The probiotic and placebo drinks were identical in packaging, appearance and composition, except for the presence of 6.5×10∧9 *Lc*S per 65 ml bottle of probiotic drink. Participants were supplied with refrigerated drinks every two weeks throughout the 4-month intervention period. Compliance was monitored through recovery of unused drinks at each delivery period.

### Study Design

This was a randomised, double-blind, placebo-controlled trial comparing the probiotic strain LcS with placebo in patients with SAR. The study was conducted out of the grass pollen season, between 11 September 2010 and 1 April 2011 (see Protocol S1 for details of subject selection and randomisation) in accordance with Good Clinical Practice including Research Ethics Committee (07/MRE05/45) approval and all participants gave written informed consent. The study was conducted according to the principles expressed in the Declaration of Helsinki. At first, procedures were undertaken to establish eligibility for recruitment to the study (described below). Following this, pre-intervention baseline measurements were made to document the intensity of local and systemic responses of each patient to a nasal allergen (pollen) challenge (NAC). At this time, patients were randomly allocated to either group A or group B by the study statistician, according to computer-generated randomisation code and given probiotic or placebo drink to consume daily. After 16 weeks each patient returned for post-intervention follow-up measurements.

### Subject Selection and Randomisation

Sixty volunteers with SAR to Timothy Grass pollen were recruited following screening for eligibility. Randomisation was performed by the study statistician using a computer generated code. Patients were randomised in blocks of 4 and 6 on a 1∶1 ratio with stratification for the presence/absence of asthma and presence/absence of allergy to tree pollen to receive 65 ml fermented milk drink containing 10∧9 *Lactobacillus casei* Shirota per ml or similar milk drink without Lactobacillus casei Shirota.

### Study Visits and Measurements

At least 2 weeks following the screening visit, subjects returned for baseline assessments which consisted of measurements and sample acquisition before, and 24 hours following a single-dose nasal allergen challenge. Following clinical examination, baseline total nasal symptom scores (TNSS) and peak nasal inspiratory flow (PNIF) were obtained in all patients on a 4-point Likert scale (0 = no symptoms, 3 = maximum symptoms). Patients with asthma underwent spirometry using a Microlab spirometer (Micro Medical Ltd, Rochester, Kent, UK) as per American Thoracic Society/European Respiratory Society guidelines [9] and recorded their total asthma symptom scores. Subjects then underwent an incremental nasal allergen challenge as described by Dreskin *et al.* [10] using Timothy grass (ALK-Abello Ltd, Hungerford, Berkshire, UK) [9]. This was performed to find the dose for individual study participants at which maximum symptoms occurred and which would then be used as the challenging dose in the study itself. Blood was taken for laboratory analyses. Nasal lavage was performed according to the method described by Grünberg *et al.* [11] One to two nasal mucosal scrapes were obtained using a Rhino-probe curette (Arlington Scientific Inc., Springville, Utah, USA) according to the manufacturer's instructions.

A single-dose nasal allergen challenge was undertaken using the concentration of allergen which approximated a TNSS of 4 during the incremental nasal allergen challenge. Nasal symptoms (described above) and PNIF (measured in duplicate) were recorded at 5, 10 and 30 minutes then hourly for 12 hours following the challenge. Patients with asthma recorded asthma symptoms, forced expiratory volume in 1 second (FEV1) and peak expiratory flow (PEF) at 5, 10, 30 and 60 minutes following the nasal allergen challenge. Nasal lavage was obtained 30 minutes following the challenge in all patients who then returned to the clinic 24 hours later for nasal symptom scoring, PNIF, blood sampling, nasal lavage and nasal mucosal scraping, plus asthma symptom scoring and spirometry for asthmatic patients.

The procedures were repeated identically after the patients had received 16 weeks of study intervention. Post-intervention assessments were made no later than March for participants with grass and tree pollen allergy and no later than April for those with isolated grass pollen allergy. (For detailed description see Protocol S1).

### Laboratory Analyses

Nasal lavages were collected as described above and frozen in dry ice immediately. Samples were then stored at −80°C and thawed prior to testing, Lavage volumes were noted at each time point and accounted for during analyses. Data for Eotaxin, IL-13, IL-1β, IL-4, IL-5, MIP-1α and RANTES were acquired by flow cytometry using a 7-plex cytometric bead array configuration (Becton Dickinson, Oxford, UK). TSLP was measured as a bead-based assay using Luminex instrumentation (Millipore UK Ltd, Watford, UK).

Nasal scrapes were cultured for 48 hours in Airway Epithelial Cell Growth Medium (Promocell, Heidelberg, Germany). Culture supernatants were removed and stored at −80°C for soluble factor tests. The soluble cytokine receptors sCD30, sIL-1RI, sIL-4R, sIL-IRII and sTNFR1 were analysed using Milliplex multi-analyte profiling technology for Luminex instrumentation (Millipore UK Ltd, Watford, UK). After removal of supernatants, adherent and non-adherent cells were taken from culture, disaggregated and permeabilized before staining with fluorochrome-labelled antibodies. Cell surface CD86 (eBiosciences, Hatfield, UK), CD252 (Biolegend, London, UK) and intracellular cytokeratin (Beckman Coulter, High Wycombe, UK) expression were documented by flow cytometry (Beckman Coulter FC500-MPL cytometer).

Peripheral blood mononuclear cells (PBMNC) were derived from heparinised blood by density gradient centrifugation. Isolated cells were cultured at 37°C, 5% CO_2_ in air for 6 days in the absence or presence of Timothy grass pollen (*phleum pratense*, Phadia AB, Uppsala, Sweden). After this period culture supernatants were removed, aliquoted and frozen at −80°C for detection of soluble molecules. IL-4, IL-5, IL-8, IL-10, IL-12p70, IL-13, IFN-γ, TNF-α, Eotaxin, MIP-1α and RANTES were analysed as a multiplex cytometric bead array. TGF-β needed to be tested on its own. All kits were purchased from BD Biosciences, Oxford, UK and data acquired by flow cytometry (Beckman Coulter FC500 MPL instrument, UK). Soluble CD23 (sCD23; Life Technologies Ltd, Paisley, UK) was quantified by ELISA.

Serum was separated from blood, frozen shortly after collection and stored at −80C until used. Pollen-specific IgG, IgG4 and IgE were quantified using the Pharmacia ImmunoCAP 100 system.

### Statistical analysis

The primary clinical end-point was the TNSS at 10 minutes following a single-dose nasal allergen challenge. A sample size of 46 participants was calculated to provide 90% power to detect a treatment difference at a two-sided 5% significance level, if the true difference between the treatments is a TNSS of 1.5 following allergen challenge assuming a standard deviation of 1.5 [10]. Secondary endpoints were PNIF at 10 minutes, area under the curve for 12 hours following nasal allergen challenge (AUC12) for nasal symptoms scores and PNIF, phenotype of cells from nasal epithelial scrapings and peripheral blood or their secretions, as well as nasal lavage inflammatory mediator profile. Using total allergic rhinitis symptom score as the response variable and treatment, subject and time as explanatory factors, a repeated measures ANOVA was used to analyse the data including time as a random effect, also treatment group, baseline score and variables used in stratification were included as fixed effects. Similar ANOVA models were used to study the effect of the other measured response variables against treatment. No adjustments were made for baseline when no differences were noted between the four stratified groups in the study. The last number carried forward procedure was applied where there were missing data or patients failed to complete the 12 hour collection period. Laboratory data were analysed using Prism software and a two-tailed Mann-Whitney non-parametric U-test, without assumption of Gaussian distribution with 95% confidence interval (CI). Analyses were performed in a blinded fashion.

## Results

### Patient recruitment and retention

**Figure 1:**
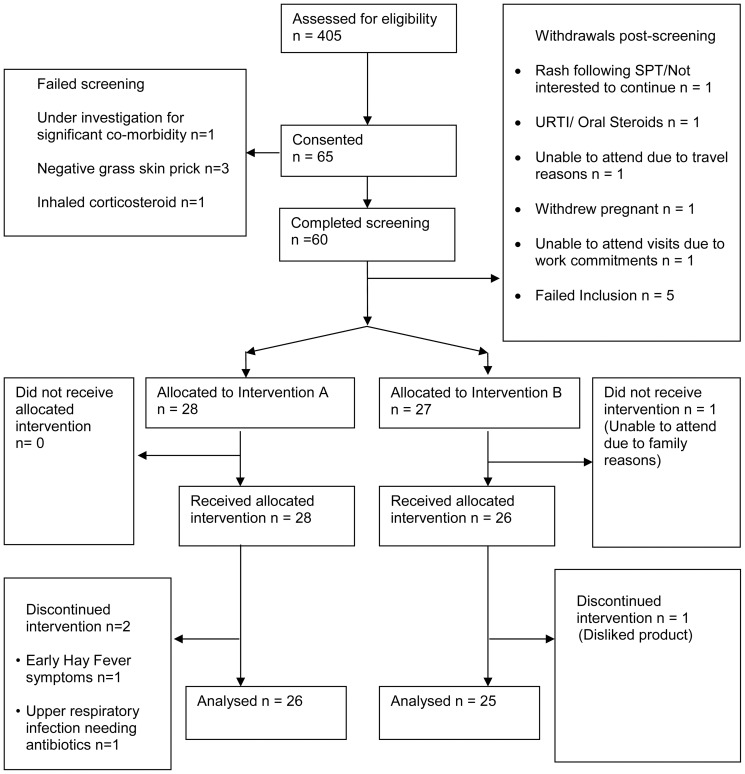
Patient recruitment and retention. Patient recruitment and retention is described.

Sixty patients entered the study of which 31 were female. They had a mean (SD) age of 42.9 (±18.6) years, mean body mass index 29.4 (±23.6) Kg/m2, mean baseline PNIF105 (±44) l/min. Fifty-five were randomised to receive treatment. They were well matched for age, gender, smoking status and baseline PNIF (Fig. 1). Patient demographics can be found in File S1.

### Rhinitis Measures at base-line and follow-up

**Figure 2:**
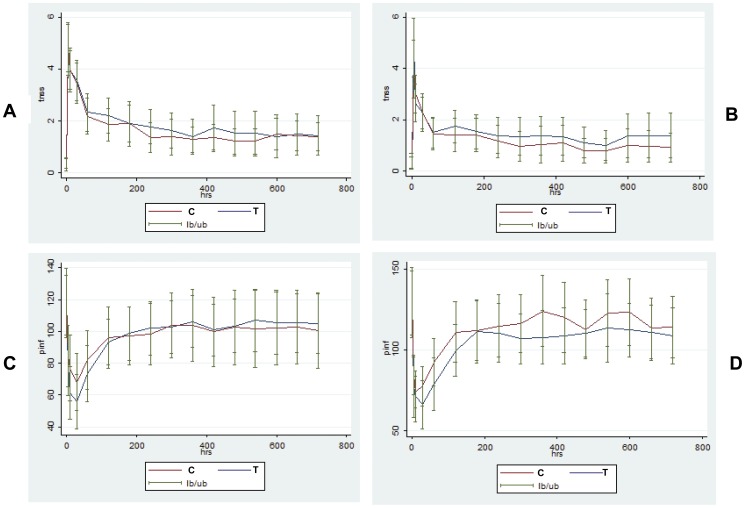
Base-line and follow-up measurements. TNSS (A,B) and PNIF (C,D) were recorded at baseline and then at 5, 10 and 30 minutes then hourly for 12 hours following nasal allergen challenge at pre-intervention (A,C) and post-intervention (B,D) periods.

A single-dose nasal allergen challenge was undertaken using the administration procedure described above. We modified the method of delivering a single nasal challenge by first determining each patient's threshold to allergen in an incremental order to predict the concentration that would cause a TNSS response of 4. We achieved a mean TNSS of 3.96 in both groups but the variance was greater than expected with some patients having a lower score than this. A minimum of 333 BAU/ml was delivered. Nasal symptoms and PNIF were recorded at 5, 10 and 30 minutes then hourly for 12 hours following the challenge. There were no differences between the two groups for TNSS, PNIF, asthma symptoms or lung function at any of the time points following the nasal allergen challenge before or after the intervention period (Fig. 2).

### Laboratory data at base-line

At baseline, there were no discernible differences between placebo (control) and probiotic (treatment) groups or any of the stratified groups for any of the parameters evaluated.

### Local effects in soluble factors at the nasal mucosa

**Figure 3:**
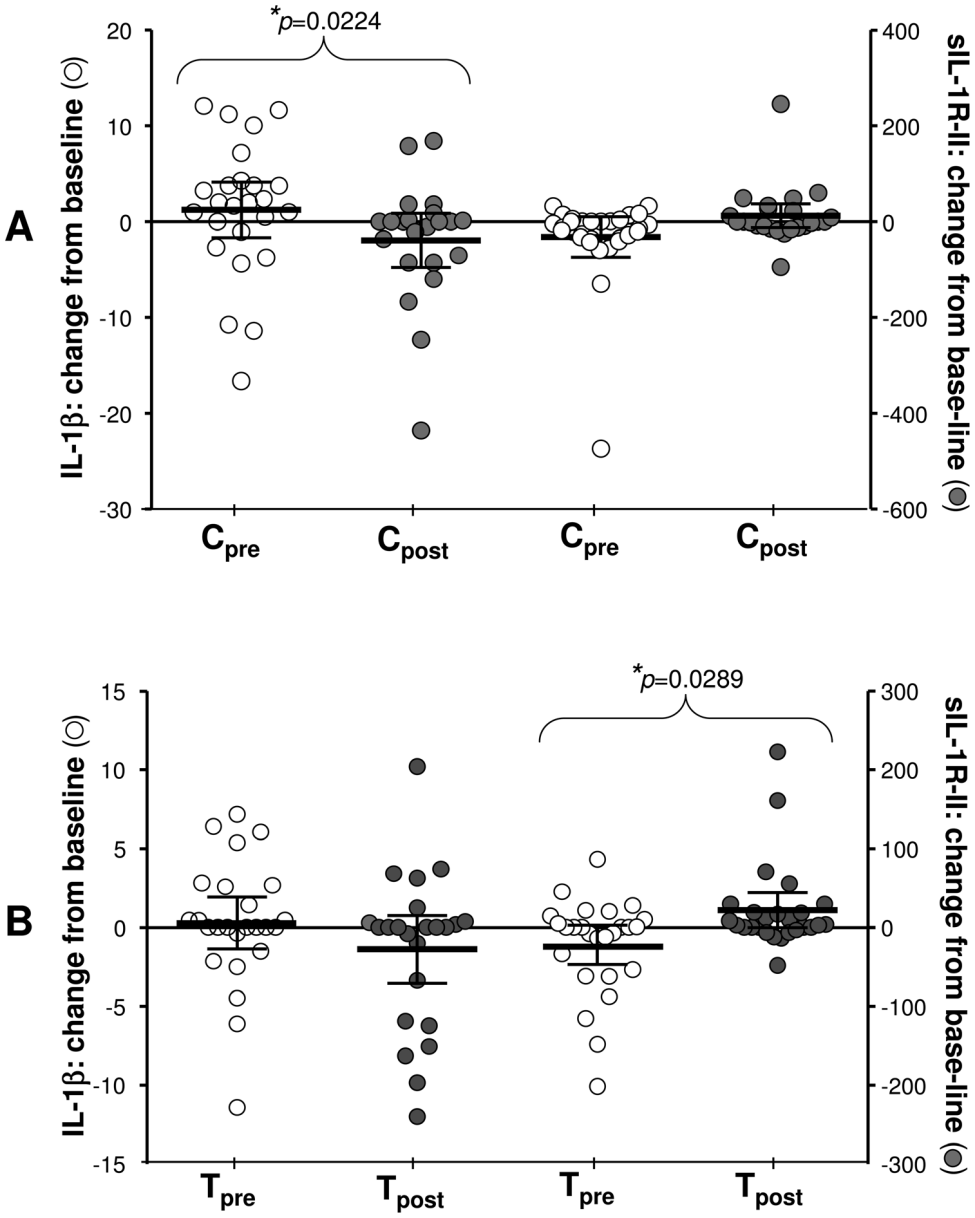
Changes in IL-1β and sIL-1RII at the nasal mucosa. Changes from base-line (24 hr–0 hr) in IL-1β levels in nasal lavage (*y*1 axis) or sIL-1RII release from nasal epithelial cells (*y*2 axis) are compared before (pre) and after (post) intervention for control (A) and treatment (B) group; (*p≤0.05). Data are presented as scatter plots showing mean pg/ml with 95% CI.

The magnitude of change at pre- and post-intervention study periods was measured by subtracting values at baseline (0 hr) from those derived after nasal allergen challenge (24 hr). This is described as change from baseline. In nasal lavage, there were no statistically significant differences between the study groups for Eotaxin, IL-13, IL-4, IL-5, MIP-1α or RANTES production (data not shown). Soluble factor receptor release from nasal turbinate cells was measured by collection of culture supernatants after their incubation for 48 hours in the absence of any further treatment. No significant changes were seen in sIL-1RI, sTNFR1, sCD30 or sIL-4R (data not shown). IL-1β levels differed between the groups at pre- and post-intervention periods in that the change from baseline was significant in the control (p≤0.0224; 95%CI; range −1.66–4.12 pre- and −4.76–0.86 post-treatment) but not the treatment group Fig. 3A,B). The mean changes were from 1.23±1.40 pg/ml to −1.95±1.35 pg/ml in the control group (Fig. 2A) and from 0.30±0.80 pg/ml to −1.33±1.00 pg/ml in the treatment group (Fig. 3B) at respective pre- and post-intervention periods. In contrast, significant change from baseline (*p* = 0.0289) in the IL-1 decoy receptor sIL-1RII, released from the cell surface by nasal turbinate cells, was apparent only in the treatment group with values of −21.97±12.08 pg/ml (95%CI range −46.85–2.91) before and 22.13±10.87 pg/ml (95% CI range −0.26–44.52) after intervention (Fig. 3B). At the same time changes from baseline in the control group were −31.81±20.39 and 12.30±11.91 before or after intervention, respectively (Fig. 3A).

### Local effects in cellular phenotypes at the nasal mucosa

**Figure 4:**
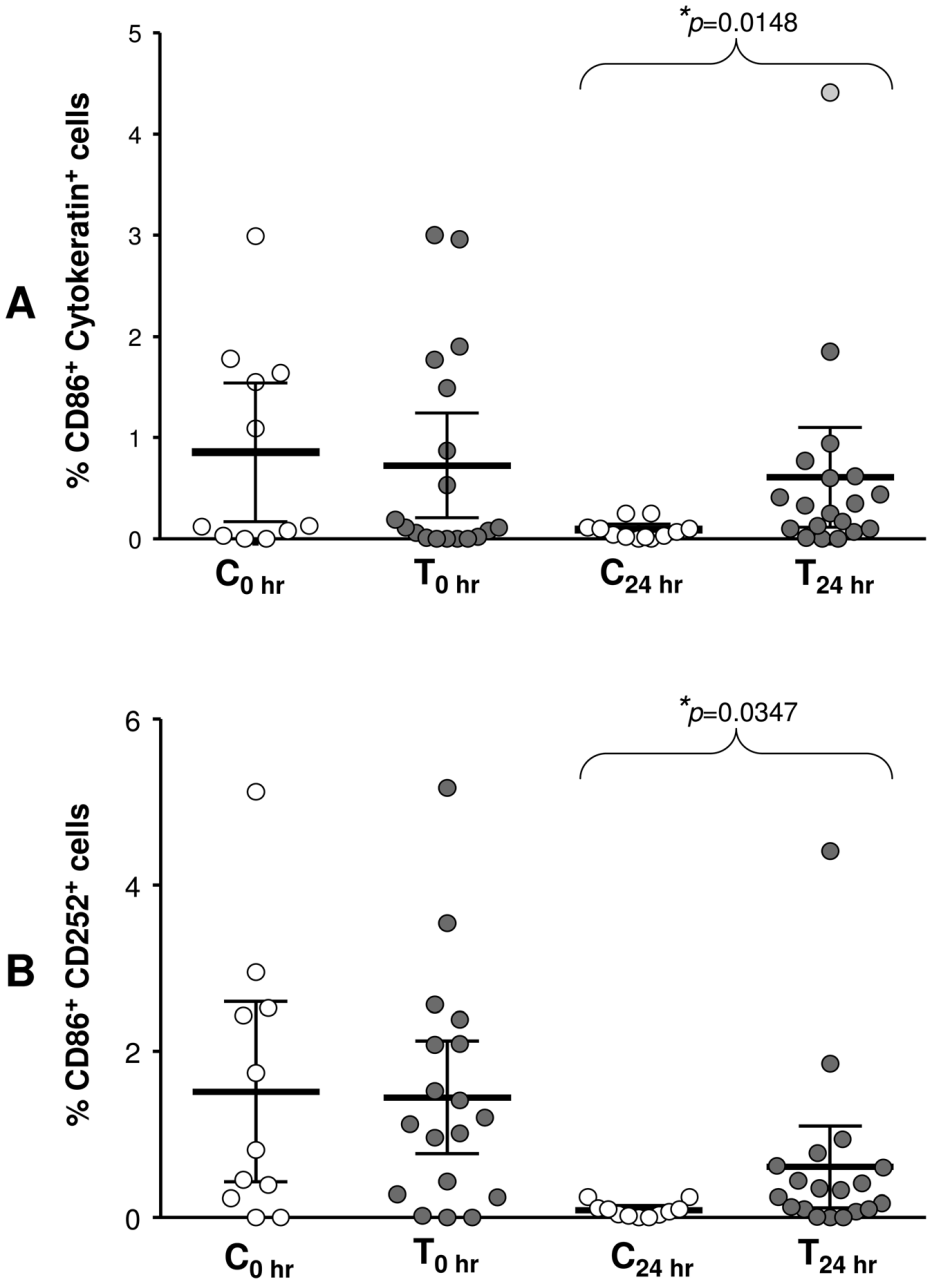
CD86 expression at the nasal mucosa. After intervention, the percentages of CD86+ Cytokeratin+ epithelial (A) and CD86+ CD252+ non-epithelial (B) cells are compared at baseline between control (C0) and treatment (T0) groups and then again 24 hours after nasal allergen challenge (C24, T24); (*p≤0.05). Data are presented as scatter plots showing mean values with 95% CI.

After collection of supernatants from nasal turbinate cells that had been cultured for 48 hr (described above), cells were collected and single cell suspensions were prepared. Post-intervention epithelial cells were identified by their cytokeratin expression and their CD86 expression compared between control and treatment groups. Constitutive expression was documented before nasal allergen challenge at 0 hr when no significant differences were apparent between control (0.86±0.31%) and treatment (1.07±0.41%) groups (Fig. 4A). When responses to allergen were measured 24 hr after nasal challenge, the control group had significantly (*p* = 0.0148) fewer CD86+ epithelial cells (0.09±0.31%; 95% Cl range 0.03–0.15) than the treatment (0.61±0.23%; 95%CI range 0.12–1.10) group. Cytokeratin negative non-epithelial cells that constitutively co-expressed CD252 with CD86 (CD86+CD252+) comprised 1.51±0.49% and 2.12±0.74% in control or treatment groups respectively (Fig. 4B). After nasal challenge the control group had significantly (*p* = 0.0347) lower numbers of CD86+CD252+ cells (0.74±0.35%; 95%CI range 0.32–1.16) than the treatment (1.78±0.19%; 95%CI range 1.05–2.51) group.

### Systemic effects in peripheral blood: cytokine release

**Figure 5:**
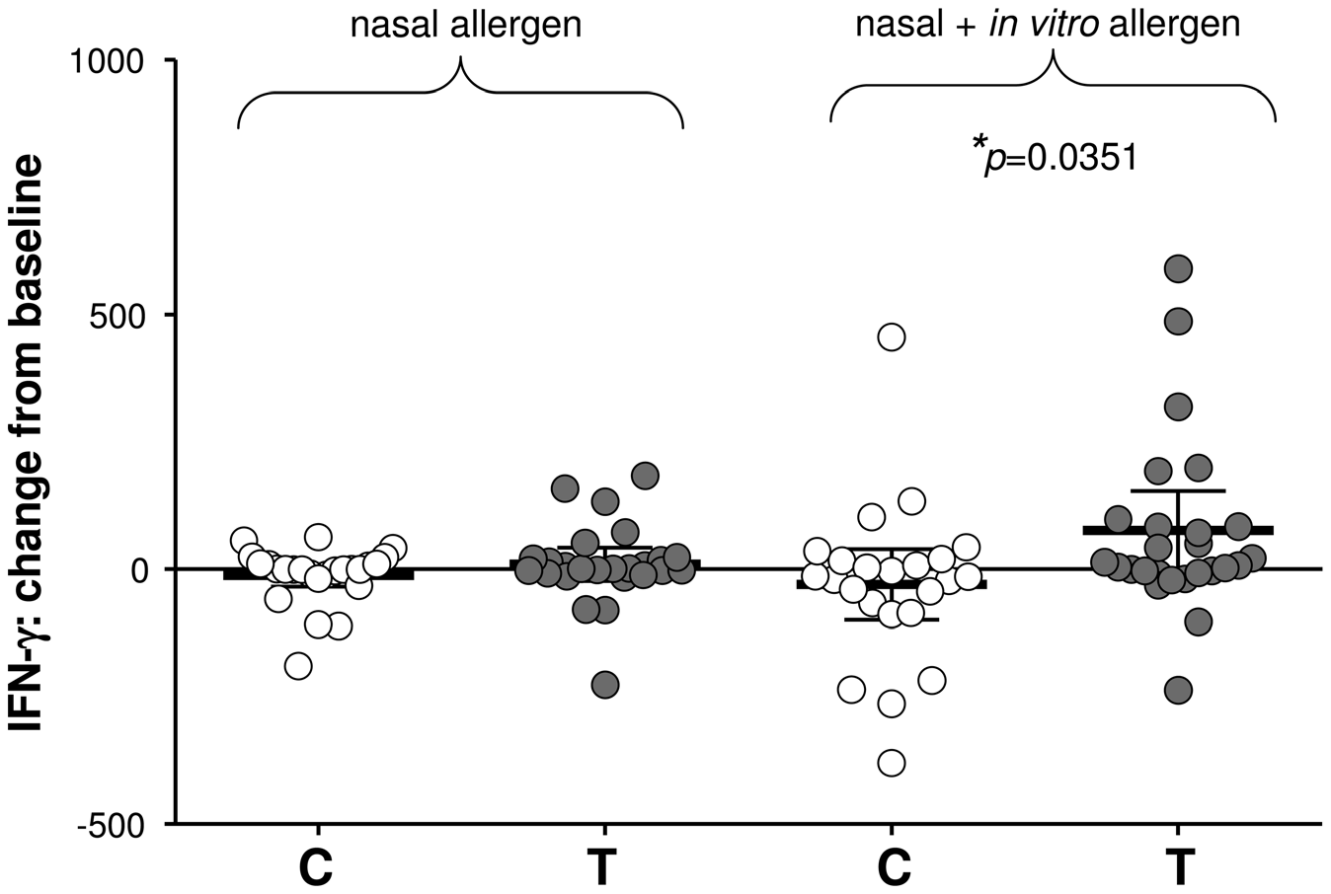
Systemic effects on IFN-γ production. Systemic effects of intervention are noted in change from baseline (24 hr–0 hr) in IFN-γ production by PBMNC cultured for 6 days in the absence (nasal allergen) or presence (nasal+*in vitro* allergen) of added pollen; (*p≤0.05). Data are presented as scatter plots showing mean pg/ml with 95% CI.

**Figure 6:**
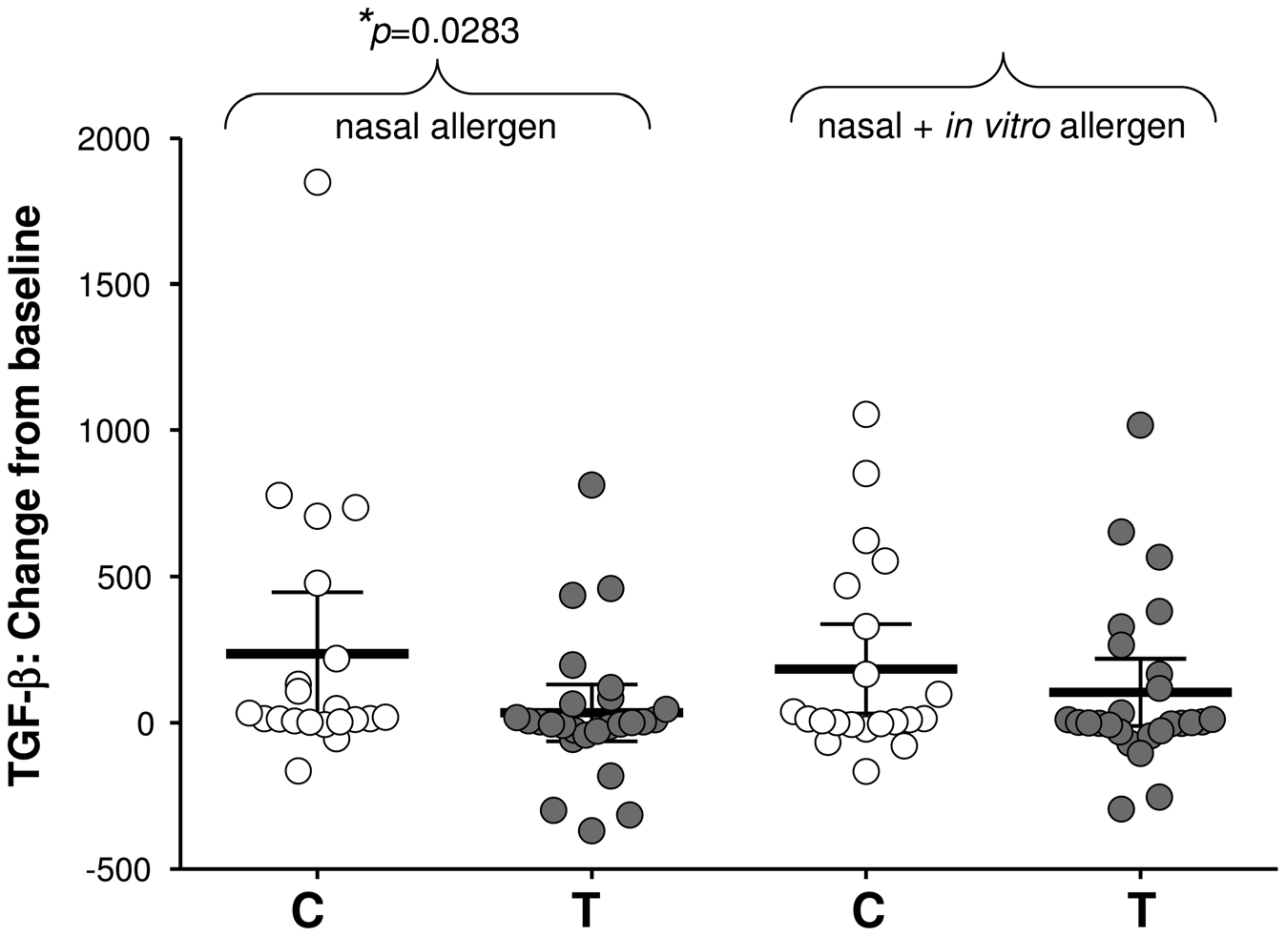
Systemic effects on TGF-β production. Systemic effects of intervention are noted in change from baseline (24 hr–0 hr) in TGF-β secretion by 6-day cultures of PBMNC in the absence (nasal allergen) or presence (nasal+*in vitro* allergen) of added pollen; (*p≤0.05). Data are presented as scatter plots showing mean pg/ml with 95% CI.

Peripheral blood mononuclear cells (PBMNC) were isolated and cultured for 6 days in the presence or absence of pollen allergen. At the pre-intervention period there were no significant differences between groups in their change from baseline after nasal challenge for any of the soluble factors tested apart from IL-8 which has been excluded from further analysis. After intervention there were no group-specific differences in the magnitude of change in IL-4, IL-5, IL-10, IL-12p70, IL-13, MIP-1α Eotaxin, RANTES or TNF-α production. The supernatants of PBMNC taken 24 hr after nasal challenge and placed in culture for 6 days gave change from baseline values in IFN-γ secretion of −11.47±10.95 pg/ml for the control group and 47.38±39.91 pg/ml for the treatment group (Fig. 5). Although more IFN-γ was produced by the treatment group, significant difference (*p* = 0.0351) was only apparent after the same cells were subjected to further *in vitro* stimulation with pollen (control −9.29±58.78 pg/ml, 95%CI range −13.09–112.30; treatment 76.81±37.82 pg/ml, 95%CI range −0.66–154.30). In contrast, a significant (*p* = 0.0283) change from baseline in TGF-β levels seen in the control group (control 253.30±100.30 pg/ml, 95%CI range 25.79–444.38; treatment 34.67±47.42 pg/ml, 95%CI range −63.00–132.30) was lost after additional *in vitro* stimulation with pollen (control 184.60±72.66 pg/ml.; treatment 103.90±55.96 pg/ml; Fig. 6).

### Systemic effects in peripheral blood: sCD23 release from the cell surface

**Figure 7:**
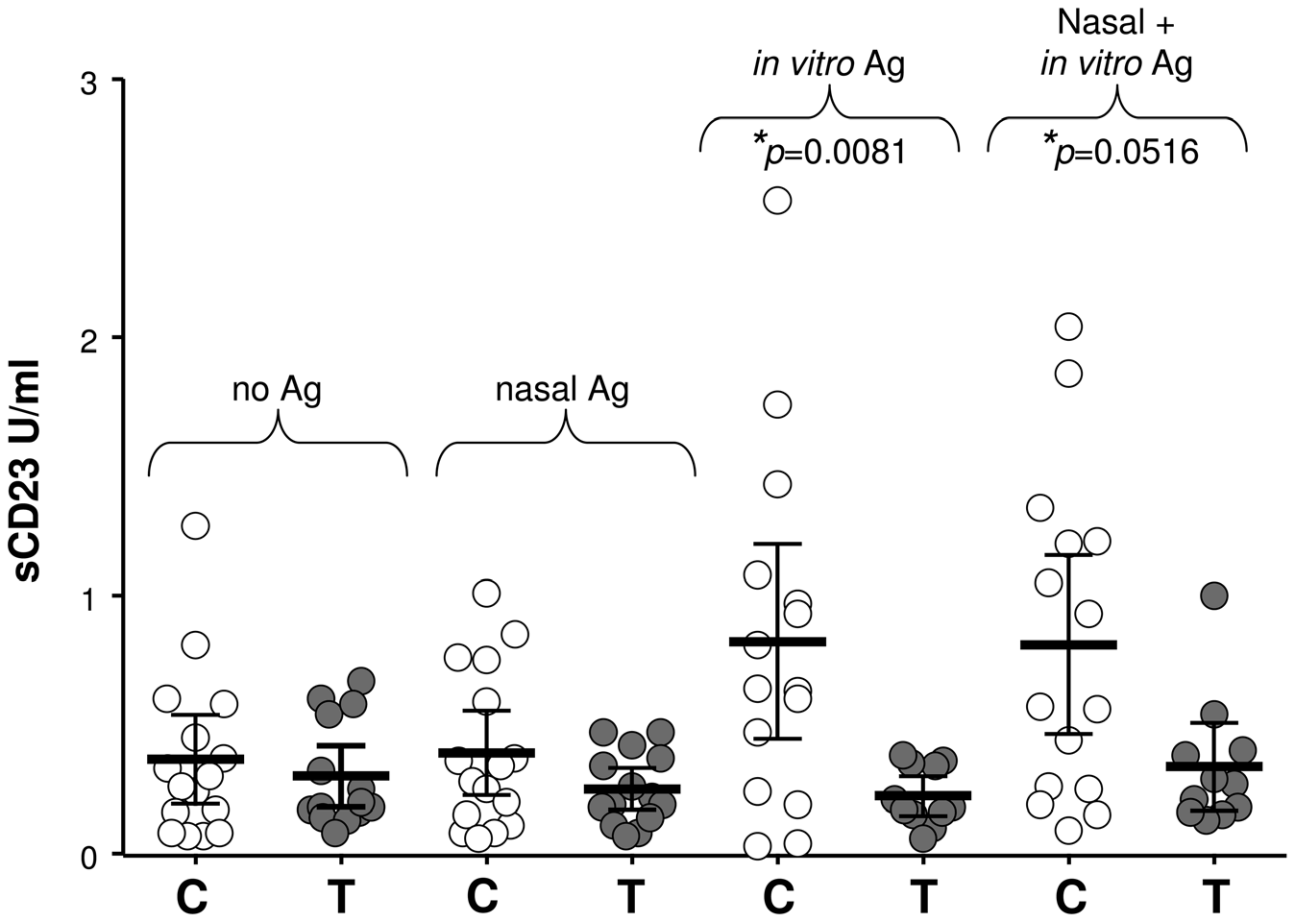
Systemic effects on sCD23 release. Systemic effects of intervention on sCD23 release from the cell surface are compared between control (C) and treatment (T) groups when PBMNC were cultured for 6 days without any challenge (no Ag), after nasal challenge (nasal Ag), *in vitro* stimulation with pollen (*in vitro* Ag) or both nasal and *in vitro* challenge (nasal+*in vitro* Ag); (*p≤0.05). Data are presented as scatter plots showing mean pg/ml with 95% CI.

As described earlier, isolated PBMNC were cultured for 6 days in the presence or absence of pollen. No differences were apparent between the groups before the intervention period (data not shown). After intervention, in the absence of any challenge constitutive release of sCD23 was 0.37±0.08 U/ml in the control group and 0.30±0.06 U/ml in the treatment group (Fig. 7). Nasal challenge had little effect, with values of 0.39±0.08 U/ml and 0.25±0.04 U/ml respectively for control or treatment group. *In vitro* addition of pollen to the cultures resulted in significantly (*p* = 0.0081) more sCD23 release by the control (0.82±0.18 U/ml' 95%CI range 0.45–1.20) compared with the treatment (0.22±0.03 U/ml, 95%CI range 0.15–0.30) group. Prior pollen challenge at the nasal mucosa had little effect on this, with 0.81±0.16 U/ml sCD23 shed by the control group and 0.34±0.08 U/ml released by the treatment group when the cells were subjected to both nasal and *in vitro* challenge.

### Serum Immunoglobulins

Next we evaluated levels of IgG, IgG4 and IgE by using Pharmacia ImmunoCAP 100 system. In contrast to what we observed during a previous study that involved a natural allergen exposure [4] no group-specific, statistically significant changes were detected in pollen-specific total IgG, IgG4 or IgE in serum. This suggested that a single nasal challenge does not accurately represent natural allergen exposure which occurs daily, at variable/lower dose during the pollen season.

## Discussion

Here we report that daily oral supplementation with *Lc*S modified some parameters of allergic inflammation at the nasal mucosa but this was not paralleled by significant changes of clinical symptoms of SAR. IL-1β release was significantly lower (*p* = <0.05) in nasal lavage fluids of the control group after allergen challenge while remaining relatively unchanged in the treatment group. Following nasal challenge, levels of IL-1β normally increase but return to basal levels within 24 hours through sequestration by IL-1 responsive cells and normal clearance mechanisms. Interestingly, the treatment group showed a higher level of sIL-1RII. IL-1RII can act as an anti-inflammatory protein that functions as a decoy receptor by binding the pro-inflammatory cytokine IL-1 [12] produced by nasal epithelial cells [13]. IL-1 can enhance mast cell cytokine secretion and histamine release and studies in mice suggest its contribution to the pathogenesis of allergic rhinitis [14]. IL-1 and IL-1R regulation may therefore be an important contributory factor to the inflammation that occurs with allergen exposure [15]. It has been shown that soluble IL-1RII binds IL-1β to generate a composite binding surface to recruit soluble IL-1 receptor accessory protein (sIL-1RAcP) that serves to increase the affinity of sIL-1RII for IL-1β [16] while also stabilizing the [IL-1+IL-1RII] complex [17]. In that case, IL-1 would be prevented from participating in the proinflammatory response and its kinetics of degradation would also be altered resulting in its prolonged detection.

The presence of IL-1β in nasal lavage before allergen challenge indicates its constitutive production in our allergic subjects, as described elsewhere [14]. Similarly, CD86+ cells were constitutively present in our nasal mucosal scrapes, possibly through upregulation by IL-1β [18]. The percentages of CD86+ cells that we detected at the epithelial mucosa were relatively small, and lower than those described by others [20], a discrepancy that we believe can be attributed to the more stringent flow cytometry gating strategy we adopted. To date, few studies have looked at co-stimulatory molecules on mucosal surfaces but an investigation using a single-cell suspension from freshly resected human inferior nasal turbinates of a healthy individual found only 0.02% of CD86+ dendritic cells [19], considerably less than were detected in our study. Viewed in the context of the whole nasal mucosa, small percentages may represent a sizeable total cell count. Indeed, it is known that small scale events taking place at the mucosal interface, such as direct dendritic cell sampling, play a major role in shaping the local immunological microenvironment [20]. A lower expression of CD86 in our control group can be explained by the work of Qureshi and colleagues [21] who show that T cells typically infiltrate the mucosa during an allergic response. On their activation, CD86 molecules on the plasma membrane of APCs co-localize with CTLA-4 on T cells and are then transendocytosed into the T cells with subsequent loss from the surface of APCs, an event of clear immunological relevance. Although we did not test for the presence of T cells or their expression of CTLA-4, a failure by the treatment group to alter their CD86 expression following nasal pollen challenge is consistent with the above observations. It also concurs with the study of Rasche and her colleagues who found higher CD86 expression after co-stimulation of PBMNC with allergen plus *Lactobacillus* [22].

As in Rasche's study, we observed that the culture supernatants from *in vitro* challenged PBMNC had lower levels of sCD23 in the treatment compared with control group. Due to the multiple forms of CD23, its many ligands and various activities of the different complexes, the mechanisms involved in Th2 regulation by CD23 are not fully understood. Although the soluble form of CD23 (sCD23) can both enhance and inhibit Th2-type responses, serum levels of this molecule are higher in patients suffering from allergic disorders [23]. A recent study has shown that sCD23 can amplify Th2 responses and that this effect depends on cleavage of membrane-bound CD23 from the cell surface [24].

sCD23 has been shown to be regulated by IFN-γ and in our study lower levels of sCD23 in the treatment group were accompanied by their higher levels of IFN-γ, associated with abated Th2 reactions. This is consistent with findings from *in vitro* studies of Roever and colleagues [25] who showed that the addition of neutralizing anti-IFN-γ antibodies increased CD23 expression on B cells stimulated with IL-4 and Birch Pollen Allergen. *In vivo*, pre-seasonal use of specific immunotherapy (SIT) has resulted in a higher frequency of IFN-γ-producing cells with improvement of both symptoms and medication use during the pollen season [26]. In addition, a reversal of established Th2 allergic responses has been shown to occur by an IFN-γ-dependent mechanism acting both locally and systemically [27], [28].

Studies of oral tolerance and experimental colitis indicate that reciprocal IFN-γ and TGF-β responses regulate the occurrence of mucosal inflammation [29]. It is also known that TGF-β can inhibit IFN-γ production and vice-versa [30], [31]. This would support the findings of our study where lower TGF-β production within the treatment group concurred with their higher levels of IFN-γ. In patients with allergic rhinitis, it is known that the production of TGF-β1 drives airway remodelling and facilitates on-going inflammation [32]. Evidence has also been provided for a correlation between TGF-β and higher intraepithelial mast cell numbers with co-localised TGF-beta receptors, suggesting that the expression of TGF-β may represent an important biological process involved in either the recruitment or retention of mast cells within the epithelium in naturally occurring allergic rhinitis [33], [34], [35]. While all of the above point towards a benefit of lower TGF-β levels in allergic rhinitis, pollen-specific immunotherapy significantly increases serum TGF-β levels that are correlated with higher IgA synthesis [36], down-regulation of Th2 responses [37] and prevention of eosinophilia and inflammation [38]. Thus, the regulatory role of TGF-β is both pleiotropic and complex and the significance of its fluctuating levels needs to be evaluated in the context of other immunological, clinical and environmental factors.

Our study shows that the interaction of probiotic organisms with the intestinal mucosa affects the nasal mucosa. To our knowledge this is the first time such an event has been documented. The biological relevance of this finding lies in the notion that events at mucosal surfaces have a systemic element to them, as evidenced by changes in blood seen in our study after nasal allergen challenge and these could influence the ensuing immune response. Indeed, a single bronchial allergen challenge with House Dust Mite (HDM) is accompanied by increased levels of allergen-specific IgE for HDM in serum and an enhanced Th2 response to HDM still detectable 5 weeks after challenge [39].

In our single-dose nasal allergen challenge model we did not detect any clinically meaningful or statistically significant benefit in terms of nasal symptoms or nasal inspiratory flow following 16 weeks of probiotic consumption. There were no significant differences either in asthma symptoms or lung function when compared with pretreatment measures. In addition to this, our study did not show any effect in terms of asthma symptoms or lung function. This result is in keeping with an earlier study with *Lactobacillus* species where no effect was noted in clinical outcomes [40], although our study included a small number of asthmatic patients all of whom had mild symptoms only. In agreement with an absence of statistically significant changes in clinical parameters, there were no group-specific differences in the laboratory analyses of classically allergy-associated components such as IL-4, IL-5, IL-13, TSLP or pollen-specific Igs, tested either in serum, nasal lavage, epithelial cell secretions or peripheral blood.

Lack of changes in the clinical symptoms could be explained by an inability of a single nasal allergen challenge to reproduce the chronicity of a natural allergen exposure. Although this approach has the advantage of overcoming the variable nature of pollen exposure [41], it does not represent a real-life situation where individuals are exposed to lower concentrations of allergen over a prolonged period of time. In our study, differences in responses seen to nasal challenge alone and those following further *in vitro* challenge suggests that both allergen dose and multiplicity of challenge can influence the outcome. The need to find a model closer to natural allergen exposure has led to the development of strategies consisting of performing daily challenges with the chosen allergen and to repeat the process over a few consecutive days. However, even this does not mimic natural exposure to allergen since it does not induce lower airway inflammation that is commonly found in rhinitis subjects naturally exposed to allergen during the pollen season [42].

We interpreted these data as showing that the interaction of probiotic organisms with the intestinal mucosa can have effects at a distant mucosal site, the nasal mucosa and suggest that single nasal allergen challenges, while permitting control of allergen exposure, may not replicate natural exposure to allergen.

## Supporting Information

Checklist S1 **CONSORT Checklist.**
(DOCX)

File S1 **Table S1: Demographics of 55 patients who were randomised to receive study medication.**
(DOCX)

File S2 **Subject Consent form.**
(DOC)

File S3 **Ethics submission.**
(PDF)

File S4 **Clinical trial registration information.**
(PDF)

Protocol S1 **Study protocol.**
(DOCX)
