# Supplementary material for: Oral Delivery of a Probiotic Induced Changes at the Nasal Mucosa of Seasonal Allergic Rhinitis Subjects after Local Allergen Challenge: A Randomised Clinical Trial
Source: PLoS One. 2013 Nov 15;8(11):e78650. doi: 10.1371/journal.pone.0078650 (PMC3829814; doi:10.1371/journal.pone.0078650)
Supplement: File S1 — Table S1: Demographics of 55 patients who were randomised to receive study medication. (DOCX) [file pone.0078650.s002.docx]

**Supporting Information S1**

*Table S1*

*Demographics of 55 patients who were randomised to receive study medication*

|  | **Probiotic**  **N** | **Mean (SD)** | **Placebo**  **N** | **Mean (SD)** |
| --- | --- | --- | --- | --- |
| Age | 28 | 42.2 (18.4) | 27 | 41.37 (18.07) |
| Female |  | 12/28 |  | 16/27 |
| BMI | 27 | 24.9 (3.97) | 27 | 28.41 (8.95) |
| PNIF | 28 | 115.79 (49.7) | 27 | 118.65 (53.79) |
| Predicted FEV_1_ | 6 | 100.5 (22.64) | 5 | 91.4 (22.19) |
| Smoking Status | 28 |  | 27 |  |
| Never Smoked |  | 20/28 |  | 14/27 |
| Ex Smoker |  | 8/28 |  | 13/27 |
| Current Smoker |  | 0 |  | 0 |
| **Strata** |  |  |  |  |
| asthma no tree allergy |  | 4/28 |  | 4/27 |
| asthma tree allergy |  | 2/28 |  | 1/27 |
| no asthma but tree allergy |  | 11/28 |  | 9/27 |
| no asthma no tree allergy |  | 11/28 |  | 13/27 |

BMI = body mass index

PNIF= peak nasal inspiratory flow

FEV1 = forced expiratory volume in 1 second.

There were clinically meaningful differences between average values for patients randomised to group A or B.
