# Supplementary material for: Oral Delivery of a Probiotic Induced Changes at the Nasal Mucosa of Seasonal Allergic Rhinitis Subjects after Local Allergen Challenge: A Randomised Clinical Trial
Source: PLoS One. 2013 Nov 15;8(11):e78650. doi: 10.1371/journal.pone.0078650 (PMC3829814; doi:10.1371/journal.pone.0078650)
Supplement: File S2 — Subject Consent form. (DOC) [file pone.0078650.s003.doc]

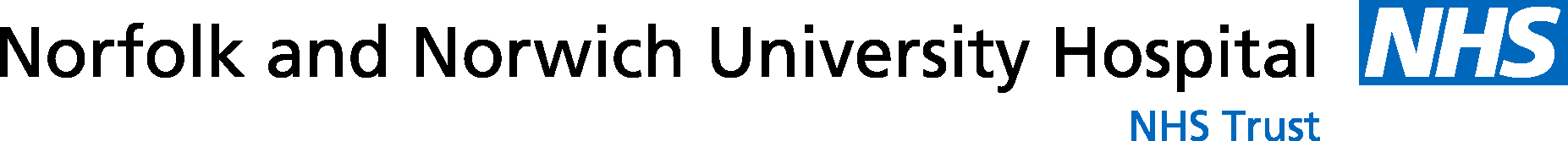


Supporting Information S2

Department of Respiratory Medicine

Level 3, East Block

Colney Lane

Norwich

# NR4 7UY

**SUBJECT CONSENT FORM**

Subject Identification Number for this trial:

**Title of Project: Evaluation of the effect of Yakult© on symptoms of subjects suffering Seasonal Allergic Rhinitis (SAR) [Rhinitis 2]**

Short title: Outcome of probiotic consumption for seasonal allergic rhinitis

**Name of Researcher**: Dr Andrew M Wilson

**Please initial box**

1. I confirm that I have read and understand the information sheet dated (………………. ⬜

Version ) for the above study and have had the opportunity to ask questions.

2. I understand that my participation is voluntary and that I am free to withdraw at any time, ⬜ without giving any reason, without my medical care or legal rights being affected.

3 I give permission for my General Practitioner to be informed about my participation in the ⬜

study

4. I understand that sections of any of my medical notes may be looked at by responsible ⬜

individuals from regulatory authorities where it is relevant to my taking part in research.

I give permission for these individuals to have access to my records.

5. I agree for my data to be stored securely in an anonymised form ⬜

6. I agree for my nasal and blood samples to be stored securely in an anoymised form for the ⬜

duration of the study pending ethical approval for another study. My samples will only be analysed

for chemicals other than those described in the patient information sheet if I provide specific consent

in the future.

7. I agree to take part in the above study. ⬜

8. I confirm that I am not pregnant, am not planning to become pregnant during the

period of the trial, and that if I become pregnant I will notify you and be withdrawn from the

trial (female subjects only) ⬜

________________________ ________________ ____________________

Name of Patient Date Signature

_________________________ ________________ ____________________

Name of Person taking consent Date Signature

(if different from researcher)

_________________________ ________________ ____________________

Researcher Date Signature

1 for patient; 1 for researcher; 1 to be kept with hospital notes
