# Supplementary material for: Oral Delivery of a Probiotic Induced Changes at the Nasal Mucosa of Seasonal Allergic Rhinitis Subjects after Local Allergen Challenge: A Randomised Clinical Trial
Source: PLoS One. 2013 Nov 15;8(11):e78650. doi: 10.1371/journal.pone.0078650 (PMC3829814; doi:10.1371/journal.pone.0078650)
Supplement: File S3 — Ethics submission. (PDF) [file pone.0078650.s004.pdf]

CONFIDENTIAL

Norfolk and Norwich University Hospital
NHS Trust

PROTOCOL SUMMARY

**Title:**	**Evaluation of the effect of yoghurt-type drink on symptoms of subjects suffering Seasonal Allergic Rhinitis (SAR)** [Rhinitis 2]
**Study sponsor**	University of East Anglia<br>Sponsor's Representative<br>Mrs Sue Steel<br>Research Contracts Manager<br>Research, Enterprise & Engagement Office,<br>The Registry, University of East Anglia,<br>Norwich, NR4 7TJ.
**Study funder**	Yakult Honsha Co. Ltd.<br>1-19, 1-chome, Higashi-Shimbashi<br>Minato-ku 05-8660, Tokyo, JAPAN<br>Contact person: Mr Oosawa
**Chief Investigator:**	Dr Andrew Wilson<br>Clinical Senior Lecturer, University East Anglia and Norfolk and Norwich NHS Hospital Trusts<br>Honorary Consultant Physician in Respiratory Medicine, Norfolk and Norwich University Hospital, Colney Lane, Norwich, NR4 7UY<br>Tel: 01603 591257<br>Fax: 01603 458553
**Principal Investigator:**	Dr Kamal Ivory<br>Senior Scientist<br>Gastrointestinal Biology and Health Programme<br>Institute of Food Research,<br>Norwich Research Park,<br>Norwich NR4 7UY<br>kamal.ivory@bbsrc.ac.uk<br>Tel: 01603 255374<br>Fax: 01603 507723
**Co-Investigator**	Professor Claudio Nicoletti<br>Gastrointestinal Biology and Health Programme<br>Institute of Food Research, Norwich Research Park, Norwich NR4 7UA, UK<br>claudio.nicoletti@bbsrc.ac.uk; Web: www.ifr.ac.uk<br>Tel: 01603 255233<br>Fax: 01603 507723

Co-Investigator: Dr Darren Sexton
Lecturer in immunology and LTN Business Fellow,
University of East Anglia, Norwich NR4 7TJ, UK
d.sexton@uea.ac.uk
Tel: 01603 593321
Fax:01603 458553

Yakult UK contact: Dr Linda Thomas
Science Manager, Yakult UK Ltd,
Artemis, Odyssey Business Park, West End Road, South Ruislip, Middlesex HA4 6QF, UK
Tel: 0208842617. Mobile: 07968 175903
Fax: 02088393250
LThomas@yakult.co.uk

Yakult Europe contact: Dr. Jia Zhao
Science Manager, Yakult Europe B.V.
Schutsluisweg 1, 1332 EN ALMERE, The Netherlands
Tel: +31(0) 36 521 1356
Fax: +31 (0) 36 532 9840
JZhao@yakulteurope.com

Protocol Synopsis

Primary objective To assess the clinical and biological efficacy of *Lactobacillus casei* Shirota (LcS) in modulating the severity of seasonal allergic rhinitis (SAR) when consumed daily over a period of 4 months

Rationale It is hypothesised that modulation of the intestinal microbiota by a daily LcS supplement as Yakult fermented milk, can modulate the immune response of SAR subjects and alleviate rhinitis symptoms. This hypothesis is supported by the findings of a previous double-blind, placebo-controlled pilot study, showing immune modulation in SAR sufferers drinking Yakult.

Study design Randomized, double-blind placebo-controlled trial measuring clinical outcomes of volunteers suffering from SAR.

Intervention Subjects will receive one bottle of Yakult containing the probiotic bacterium *Lactobacillus casei* Shirota (active drink) or a similar-looking placebo, once daily for 4 months (16 weeks).

Site and subjects 60 adult subjects (30 in each of two groups). All study procedures involving participants will be conducted within the Clinical Research Trials Unit (CRTU) at the Norfolk and Norwich University Hospital (NNUH). Samples taken will be processed at both UEA and IFR. Inclusion/exclusion criteria are detailed below. Summarised: men and women >16 years of age with a history of seasonal allergic rhinitis.

Procedures The study aims to examine the effect of probiotics on the clinical symptoms of allergic rhinitis and to elucidate some of the immunological mechanisms involved. Following a screening procedure, enrolled participants will undergo nasal allergen challenge when their baseline clinical and immunological responses will be measured. Participants will then be randomized on a 1:1 ratio to receive probiotic or placebo formulation. They will consume these for a period of 4 months after which they will return for a further nasal allergen challenge and post-treatment clinical and laboratory assessment of responses.

Primary endpoints Total nasal symptom score (TNSS) 10 min after nasal allergen challenge.

Secondary endpoints

- Area under the curve for nasal symptoms scores for 12 hours following nasal allergen challenge
- Area under the curve for peak nasal inspiratory flow for 12 hours following nasal allergen challenge
- Phenotype of nasal epithelial cells from scrapings
- Nasal lavage inflammatory mediator profile

Statistical analysis Sample size calculated based on 46 participants providing 90% power to detect a treatment difference at a two sided 5% significance level, if the true difference between the treatments is a TNSS of 1.5 following allergen challenge based on the assumption that the standard deviation of TNSS following nasal allergen challenge is 1.5 (Dreskin *et al* 2002). Sixty participants will be enrolled to allow for 20% drop-out.

1. Introduction and background

Epidemiological studies have shown that the incidence of atopic diseases (eczema, food allergy, allergic rhinitis and asthma) has been rising over the last few decades. The rate of increase precludes genetic make-up as the sole cause of the atopic epidemic and implicates environmental factors instead. Currently, allergic rhinitis (hay fever) is one of the world's most common chronic allergic diseases. It affects over 600 million people and often leads to asthma. There are huge costs associated with the condition in terms of both health care and work days lost, with British businesses estimated to lose £324 million this summer alone. For the sufferers, the symptoms severely affect their quality of life. It disturbs their sleep and impairs daytime concentration and performance at work or school. Currently there is no cure for it.

The importance of the gut microbiota in general well-being is evidenced by several experimental observations. It is difficult to achieve oral tolerance in germ-free animals [Sudo *et al.* 1997] while administration of lipopolysaccharide (a constituent of the outer membrane of gram-negative bacteria) together with food antigens increases the tolerizing effect of feeding [Kim & Ohsawa 1995]. While able to assist in tolerance induction, bacterial products may also break oral tolerance [Gaboriau-Routhiau *et al.* 1996]. These findings led Wold to suggest in 1998 that an altered normal intestinal colonization pattern in infancy, which fails to induce immunological tolerance, could be responsible for the increase in allergies. Recent studies suggest that the effects of the gut microbiota may not only be related to food antigens, but also to aeroallergens [Noverr *et al*. 2004, 2005]. Forsythe and his colleagues (2007) managed to attenuate adverse airway responses in a mouse model of allergic asthma through oral administration of probiotics.

The mechanisms by which probiotics exert their effects are unknown at present, but experiments in mice have documented improved gastrointestinal barrier function [Ewaschuk *et al.* 2008]. Since these experiments have also revealed a strain-dependent heterogeneity in the efficacy of probiotics [N.G. Hord 2008] it is unlikely that improved barrier function alone is responsible for the beneficial effects noted. Given the sheer numbers of microorganisms that inhabit our mucosal surfaces, it is likely that there are normally bidirectional interactions between them and the epithelial, immune, neurologic and endocrine physiological processes initiated by and between them. We hypothesize that a probiotic organism is ingested in sufficient quantities to amplify its particular trait relative to the milieu of other organisms present in relatively lower quantities, temporarily over-riding the diversity present. In that case, the transfer of information between the probiotic organism and cellular components of the gut has particular impact. In order to understand and manipulate this probiotic-mucosa cross-talk towards therapeutic advantage, there is a need to focus on the transfer of information between the microbiota and cellular components of the mucosal immune system.

Few studies have examined the effect of probiotics on allergic rhinitis and the studies that have been performed [Helin *et al*. 2002; Wang *et al*. 2004; Xiao *et al*. 2006] are inconclusive. In our own pilot study we tested the ability of *Lactobacillus casei* Shirota (LcS) to alter immunological events in seasonal allergic rhinitis (SAR) [Ivory *et al*. 2008]. The study format was double-blinded and placebo-controlled with ten SAR sufferers in each group. We compared changes in immune status arising through the daily ingestion of a milk drink supplied by Yakult, with or without live LcS, over a period of 5 months. Pre-, peak- and post-grass pollen season blood samples were collected for determination of plasma grass pollen-specific IgG and IgE levels by immunoassay. At the same time, cytokine levels were determined by flow cytometric bead array technology following culture of peripheral blood mononuclear cells for six days in the presence or absence of specific grass pollen antigens. We found that volunteers treated with LcS showed a significant reduction in levels of antigen-induced IL-5, IL-6 and IFN-γ production compared to volunteers supplemented with

placebo. Meanwhile, levels of pollen-specific IgG increased and IgE decreased in the probiotic group. Other changes in cytokine levels were seen but they did not attain statistical significance, most likely due to the small number of volunteers tested. Our work has established for the first time that probiotic supplementation modulates immune responses in allergic rhinitis through down-regulation of both Th1- and Th2-type cytokines and to beneficially alter the balance of pollen-specific IgG and IgE levels in allergic rhinitis subjects. It has also suggested that the impact of probiotics reaches beyond the intestine.

We would now like to conduct another study to show that the immunological changes arising through probiotic consumption have an impact on the clinical symptoms of hay fever. In addition, we propose to elucidate some of the mechanisms involved that may contribute to the health benefit. As far as we know there have not been any similar studies to date. The ability to demonstrate clinical efficacy of dietary intervention for hay fever treatment has obvious benefits for the relevant sufferers. Economic benefits would arise through the relatively inexpensive 'treatment' that would be self-administered and likely lower absenteeism from work. There is also a need for evidence-based viability for the many health claims made for probiotic consumption that could result in clear guidance to the general public.

2. Rationale

The bacteria present in the gut outnumber all the cells in the human host by more than ten to one. At the same time, immune tissue associated with the human gut constitutes about 80% of all immunologically active cells in the body. The ability of the gut to accommodate both is not an accident of nature but arises by design as cells of the gut interact with intestinal bacteria, both resident and ingested, to develop protective mechanisms by improving gut barrier function and immune stimulation for defence. This symbiotic relationship is also responsible for appropriate, non-exaggerated responses through immune modulation and immune tolerance [MacDonald *et al*. 1994; J.M.Saavedra 2007]. Thus, the intestinal microbiota profoundly influences the development of specific and nonspecific, cellular and humoral, local and systemic mucosal immune responses.

An association between the gut microbiota and allergy was suggested two decades ago when it was noted that the composition of the intestinal microbiota is different in infants with and without eczema [Bjorksten *et al.* 1999]. More recently, involvement of the gut microbiota has been implied in the dyregulated immunity of allergy through the ability of certain probiotic organisms to avert systemic allergic symptoms in allergen-stimulated inflammatory responses in the gut [Kirjavainen *et al*. 2002]. This anti-allergic effect is associated with oral tolerance induction [Isolauri *et al*. 2000].

Direct evidence of the effect of probiotics on respiratory defence comes from a study of BALB/c mice, in which infant mice fed *L. casei* before inoculation with influenza virus fared better than their control littermates [Yasui *et al.* 2004]. Some clinical studies seem to confirm these promising findings in mice. In view of these findings, it is reasonable to suppose that probiotics could have an effective application in modulating the severity of hay fever symptoms when incorporated into the normal diet.

3. Hypothesis

The science behind the study hypothesis is explained in more detail above (2. Rationale).

The pollens that give rise to hay fever induce abnormal IgE responses associated with overproduction of Th2 type cytokines which also contribute to the eosinophilia and accompanying airways inflammation which are typical of this disease. The intestinal microbiota seems to play an important role in the Th1/Th2 balance and the use of probiotics may redress

the Th2 imbalance in hay fever. We hypothesise that beneficial modulation of the intestinal microbiota of hay fever sufferers by daily consumption of the probiotic *Lactobacillus casei* in the form of Yakult fermented milk, will alleviate the severity of their allergic rhinitis symptoms by suppression of their allergic immune response.

4. Objective

- To assess the clinical efficacy of *Lactobacillus casei* Shirota (LcS) in modulating the severity of nasal symptom scores following nasal allergen challenge when consumed daily over a period of 4 months.
- To assess the efficacy of LcS in modulating immunological parameters in nasal lavage, nasal scraping and peripheral blood following nasal allergen challenge; when consumed daily over a period of 4 months.

5. Study Design

5.1. Overview

This is a, double-blind, placebo-controlled randomised trial to compare Yakult (containing the probiotic strain *Lactobacillus casei* Shirota) with placebo. Participants will attend on 5 to 7 occasions over a 4 to 6 month period for three procedures, all of which will be conducted at a laboratory within the Clinical Trials Research Unit (CRTU) at the Norfolk and Norwich University Hospital.

Procedure 1: Establishment of study eligibility ~ participant visit 1
The first procedure will comprise a screening visit where written informed consent will be obtained and eligibility criteria will be established. Demographic status and medical history will be obtained by a researcher and documented in the case report form. Participants will undergo a clinical examination including examination of the respiratory (including anterior rhinoscopy) and cardiovascular system by a medically qualified investigator. Nasal symptom scores will be obtained and 10 ml peripheral blood taken for routine biochemistry and haematology. Participants will undergo a baseline nasal allergen challenge to determine the dose required to elicit a positive clinical reaction. Asthmatic participants will undergo spirometry. Asthma will be diagnosed as a clinical diagnosis by a medically qualified investigator based on the participants clinical history including details of participant-reported investigations and treatments. This procedure will take place between September and November.
Procedure 2: Pre-intervention baseline measurements ~ participant visits 2, 3 and 4
Procedure 2 involves participant visits on two consecutive days. This will involve nasal samples being obtained before and 24 hours after the nasal allergen challenge. At the first of the 2-day procedure (visit 2), participants will have a clinical examination, and baseline nasal and total allergic rhinitis symptoms scores, nasal peak inspiratory flow, nasal scrapings and nasal lavage. 20ml blood will be taken for laboratory analysis. They will then undergo a nasal allergen challenge and record their nasal symptoms and peak nasal inspiratory flow at 5 mins, 30 mins then hourly for 12 hours. A nasal lavage will be collected at 30 mins. Asthmatic participants will undergo spirometry prior to and 1 hour following the nasal allergen challenge. They will also record asthma symptom scores prior to the challenge and hourly following the challenge. Those participants able to return after 6-8 hours (visit 3) to give a nasal lavage, peak nasal inspiratory flow (PNIF) and asthma symptom scores (ASS) and spirometry in asthmatic participants. These subjects will form a subgroup. All participants will return for the 24 hours later (visit 4) following the nasal allergen challenge, for a second nasal scraping and lavage. 20 ml peripheral blood will be taken at this time for laboratory analysis. Following this, participants will be randomized to active drink or similar placebo daily for 4 months. Procedure 2 will take place at least 2

weeks after procedure 1 and no later than November. Participants with isolated grass pollen allergy will attend for procedure 2 no later than December 201009.

Procedure 3: Post-intervention follow-up measurements ~ participant visits 5, 6 and 7
Procedure 3 involves participant visits on two consecutive days when they will undergo follow-up assessment identical to procedure 2. Procedure 3 will take place 4 months following procedure 2 and no later than March 20101. Participants with isolated grass pollen allergy will attend for procedure 3 no later than April 20101.

5.2. **Outline of Study Procedures**

Approximate dates in the year	Sept – Dec	By end of Dec			By end Dec	By end April			By Nov 2011~~0~~
	Screening	Baseline			Intervention	Follow up			End
	Proc. 1	Proc. 2			Start	Proc. 3			Report
		Day1		Day2		Day 1		Day 2	
		0hr	8hr	24hr		0hr	8hr	24hr	
Written consent	x								
Inclusion/exclusion criteria	x								
Demographic status and medical history	x								
Clinical examination	x	x				x			
Skin prick test for common inhaled allergens	x								
Total nasal and allergic rhinitis symptom scores	x	x	x	x		x	x	x	
Peak nasal inspiratory flow	x	x	x	x		x	x	x	
Initial intranasal allergen challenge	x								
Spirometry (asthmatic participants only)	x	x	x	x		x	x	x	
Modified intranasal allergen challenge		x				x			
Active or placebo drink: 1 bottle per day for 4 months					x	x			
Nasal scraping, peripheral blood samples taken		x		x		x		x	
Lavage		x	x	x		x	x	x	
Characterisation of cells from nasal samplings		x		x		x		x	
Epithelial & peripheral blood cell culture, analysis of TSLP, cytokines, chemokines, soluble cytokine receptors, sCD23 following *in vitro* allergenic challenge		x		x		x		x	
Measurement of Eotaxin, MIP-alpha and RANTES in nasal lavage		x	x	x		x	x	x	
Differential counts of cells derived in nasal lavage		x	x	x		x	x	x	

(Proc. = Procedure)

5.2.1 Outline of Study Procedures

Procedure 1: Screening

- SPT, TNSS, PnIF
- Intranasal Challenge.
- Spirometry, ASS (in asthmatics)

↓ ***> 2 weeks***

Procedure 2: Baseline Tests

- Clinical examination, TNSS, PnIF
- Modified Intranasal Challenge, TNSS, PnIF
- Spirometry, ASS (in asthmatics)

Nasal lavage (0, 0.5, *[6-8], 24hr)

- Measure IL-1α, IL-4, IL-5, IL-13, tryptase, ECP, TSLP, SCF

*[6-8]=subgroup able to attend

Nasal Scrapings (0, 24hr)

Cytospins

- Differential count

Epithelial cell cultures

- Analysis CD80, CD86,OX40L, CD117
- CD117, IgE, tryptase, chymase
- Soluble cytokine receptors

Peripheral Blood (0, 24hr)

- Serum pollen-specific IgE, sCD23
- Stimulated cultures FceR1 on DC
- Unstimulated cultures FceR1 on Dc

↓ ***16 weeks from Base-line tests***

Procedure 3: Post-Intervention

- Clinical examination, TNSS, PnIF
- Modified Intranasal Challenge, TNSS, PnIF
- Spirometry, ASS (in asthmatics)

Nasal lavage (0, 0.5, *[6-8], 24hr)

- Measure IL-1α, IL-4, IL-5, IL-13, tryptase, ECP, TSLP, SCF

*[6-8]=subgroup able to attend

Nasal Scrapings (0, 24hr)

Cytospins

- Differential count

Epithelial cell cultures

- Analysis CD80, CD86,OX40L, CD117
- CD117, IgE, tryptase, chymase
- Soluble cytokine receptors

Peripheral Blood (0, 24hr)

- Serum pollen-specific IgE, sCD23
- Stimulated cultures FceR1 on DC
- Unstimulated cultures FceR1 on Dc

5.3. Study subjects

5.3.1. Number of Subjects/Controls

In this study, 30 participants will receive the active drink and 30 the control placebo preparation, (a similar milk drink without any probiotic bacteria).

5.3.2. Setting

All participants will attend Clinical Research Trials Unit (CRTU) at the Norfolk and Norwich University Hospital for their study procedures.

5.3.3. *Inclusion Criteria*

Participants to be included will meet the following criteria:

- Men or women >16 years of age.
- A history of SAR for a minimum of 2 years before study entry.
- Documentation of sensitivity by positive skin testing (by prick or intradermal methods) or by adequately validated in vitro tests for specific IgE (e.g., RAST, PRIST) to grass pollen within 12 months prior to enrolment. If this is not available, appropriate tests will be performed at screening.
- Able to provide written informed consent

5.3.4. *Exclusion Criteria*

The following conditions should exclude possible study participants:

- Ingestion of probiotics as part of normal diet
- Significant medical, surgical or psychiatric disease that in the opinion of the participants' attending physician would affect subject safety or influence the study outcome.
- Symptoms of rhinitis at screening indicated by total symptom scores of more than 2 out of 12 (based on a combination of nasal symptoms of blockage, sneezing, rhinorrhoea and itching).
- Current smokers or ex-smokers of <1 year or those who have smoked the equivalent of 20 cigarettes/day for 20 years or more.
- Participants receiving any form of corticosteriod from 1 month prior to the study
- Inadequate washout periods for the following:
    - Intranasal cromolyn (2 weeks)
    - Intranasal or systemic decongestants (3 days)
    - Intranasal or systemic antihistamines (3 days), except astemizole (6 weeks) or loratadine (10 days).
- Documented evidence of acute or significant chronic sinusitis
- A history of hypersensitivity to the milk or its products
- Pregnant women or those planning a pregnancy. It is important not to include pregnant women in the study due to the possibility of miscarriage following anaphylaxis.
- Lactating women are excluded as those infants breast fed by mothers responding to allergenic challenges can transmit the manifestations of allergic responses to the feeding infant via breast milk

5.4 Randomisation

Stratified randomisation will be used. Such randomisation, by asthma and isolated grass allergy, would result in 4 different randomisation lists - one for individuals without asthma and without grass allergy, one for individuals with asthma but without grass allergy, one for individuals without asthma but with grass allergy and for individuals with both asthma and grass individuals - for each of which we shall use random permutted block randomisation (block sizes 4 and 6). Although this may result in a slight imbalance in the total number of individuals randomised to yakult or

control the maximum imbalance would be 3 individuals per strata. Study recruitment will stop when 60 individuals have been randomised.

5.5 Recruitment

Up to sixty participants with seasonal allergic rhinitis (SAR), will be recruited by advertisements within the Norwich Research Park campus, through the IFR) 'Human Studies' database, Respiratory Research Database, in the local press and via local GP surgeries. This is to have a completed sample size of 46 participants and allows for a 20% drop out rate.

All volunteers will be approached by the recruiting source (e.g GPs from GP surgeries and nurses at the Human Nutrition unit of IFR). They will have participant information sheets sent to them with their letter of invitation to participate in the study. Where recruits are obtained through advertisements within the Norwich Research Park campus or local press they will be invited to make contact with the Chief or Principal Investigators and on doing so will be sent relevant information by post. Due to time constraints in starting and completing the study before the following pollen season, it will not be possible to conduct face-to-face interactions with potential participants. However, the 'Participant Information Sheet' invites interaction by telephone with Drs. Andrew Wilson or Kamal Ivory for further study details, to answer any questions or address any concerns. This will be prior to visit 1. On expression of interest in participation, potential study participants will all be directed to Dr. Andrew Wilson, the Chief Investigator. Those volunteers wanting to participate in the study will be invited to visit the study nurses at the CTRU laboratory for face-to-face encounter when they will be able to ask any further questions about the study. If interest is retained following this they will sign consent forms and have their pre-study assessments performed.

5.6 Compliance

Compliance will be monitored through recovery of unused drinks at each delivery period. All study participants will also be provided with a calendar on which to record details of their intake of study drinks.

5.7 Regulatory Approval

Yakult has food product status in the UK and therefore this trial is not subject to regulation by the MHRA as a clinical trial.

The placebo is the same milk matrix as used in Yakult but without any *Lactobacillus* culture.

5.8 Ethics Review Committee

Approval from the Human Research Governance Committee at IFR (HRGC), a local Research Ethics Committee and the National Research Ethics Service (NRES) will be obtained before the study is commenced.

5.9 Informed Consent

Written informed consent will be obtained from all participants prior to recruitment. This will be taken by the study nurses within the CTRU of the Norfolk and Norwich University Trust Hospital.

5.10 Study Sequence

5.10.1 Study Procedure 1: *participant visit 1 ~ Screening Visit*

Once informed consent is obtained, clinical history and examination will be performed and eligibility criteria will be checked. Eligible participants will be recruited

to the trial. This procedure will take place between September and December 201009. The following measurements will be performed:

- Skin Prick Allergy Test
- Routine biochemistry and haematology
- Total Nasal Symptom Scores
- Intranasal Allergen Challenge
- Spirometry (participants with asthma only)

Haematology will include haemoglobin, platelet count, white blood count and differential while blood count. Biochemistry will include renal function (Sodium, potassium, urea and creatinine) and liver function (albumin, bilirubin, alkaline phosphatase, alanine transaminase).

5.10.2 Study Procedure 2: *participant visits 2, 3 and 4 ~ Baseline tests*

Procedure 2 will take place at least 2 weeks after procedure 1, and no later than December for isolated grass pollen allergy or November for participantss with grass and tree pollen allergy. This procedure entails participant visits on two consecutive days with baseline measurements being taken initially (visit 2) and nasal samples being obtained at 6-8 hours (visit 3) from a subgroup of participants and at 24 hours (visit 4) from all participants after nasal allergen challenge.

The subjects will undergo a clinical examination. Baseline measurements will be the following:

- Total Nasal Symptom Scores
- Peak Nasal Inspiratory Flow (PnIF)
- Modified Intranasal Allergen Challenge
- Nasal lavage, nasal scrapings and peripheral blood
- Spirometry and asthma symptom scores (participants with asthma only)

Nasal lavage, nasal scraping and 20ml (approximately 4 teaspoons) peripheral blood will be obtained before and 24 hours after nasal allergen challenge. Nasal lavage will also be taken 30 minutes after allergen challenge and, in a subgroup, 6-8 hours after allergen challenge

- Characterisation of cells derived from nasal scrapings
- Epithelial and peripheral blood cell culture and analysis of TSLP, cytokines, chemokines and soluble cytokine receptors and sCD23 following *in vitro* allergenic challenge
- Eotaxin, MIP-1α and RANTES in nasal lavage
- Differential counts of cells derived in nasal lavage and nasal scrapings

Participants with isolated grass pollen allergy will attend for procedure 2 no later than December 201009.

5.10.3 *Intervention and delivery schedule*

Participants will be randomised on a 1:1 ratio to receive probiotic or placebo formulation. Samples of the milk drinks, active or placebo, will be delivered by Yakult Europe B.V to IFR Norwich every 2 weeks. The samples will be HACCP certified for the safety of materials, production process and microbiological analysis (issued by TNO, Holland). The active and placebo drinks will be identical in composition except for inclusion of 6.5x10^9 LcS organisms per 65ml of dairy drink. Volunteers will be supplied with probiotic or placebo milk drinks every 2 weeks throughout the 4-month intervention period. These samples will be distributed to the subjects by IFR Norwich.

5.10.4 *Week -4 to 0*
Subjects will be asked to refrain from consuming any probiotic products from this point onwards for the course of the whole trial

5.10.5 *Week 0*
Subjects will start a 4-month intervention period, consuming either active yoghurt-type drink or a similar placebo.

5.10.6 Study Procedure 3: *participant visits 5, 6 and 7 ~ Post-intervention assessment*
This procedure will take place 4 months (16 weeks) after procedure 2 and no later than April for isolated grass pollen allergy or March for participants with grass and tree pollen allergy. Procedure 3 will entail participant visits on two consecutive days with nasal samples being obtained before (visit 5), at 8 hours (visit 6 for those able to return) and 24 hours (visit 7) for all participants following nasal allergen challenge. Subjects will be asked to refrain from taking anti-histamines for at least 3 days before this procedure.

Post-intervention measurements will be the following:
- Total Nasal Symptom Scores
- Peak Nasal Inspiratory Flow (PnIF)
- Modified Intranasal Allergen Challenge
- Spirometry (participants with asthma only)

Nasal lavage, nasal scraping and 20 ml (~4 teaspoons) peripheral blood will be obtained before and 24 hours after nasal allergen challenge. Nasal lavage will also be taken 30 minutes after allergen challenge and, in a subgroup, 6-8 hours after allergen challenge. These samples will be used for:
- Characterisation of cells derived from nasal scrapings
- Epithelial and peripheral blood cell culture and analysis of TSLP, cytokines, chemokines and soluble cytokine receptors and sCD23 following *in vitro* allergenic challenge
- Eotaxin, MIP-1α and RANTES in nasal lavage
- Differential counts of cells derived in nasal lavage and nasal tubinates

5.11 **Administration of active and placebo drinks**
Active and placebo drinks will be supplied as fermented milk in sealed pots of 65 ml with date stamped expiry. They should be stored at approximately 7 degrees Celsius (domestic **refrigerator**).

Fresh supplies of both products will be delivered to IFR Norwich every two weeks, and participants will receive delivery every two weeks from the institute.

5.12 **Concomitant medication**
The following concomitant medications will be permitted providing sufficient 'wash-out' period is adhered to
- Intranasal cromolyn (2 weeks)
- Intranasal or systemic decongestants (3 days)
- Intranasal or systemic antihistamines (3 days)
- Antihistamines (3 days), except astemizole (6 weeks) or loratadine (10 days).

6 Study procedures

6.1 Skin prick testing

This test measures the presence of allergen-specific IgE attached to cells in the skin. It will be undertaken with a positive (histamine) and negative (saline) control and to common inhaled allergens. No antihistamines should be taken for 3 days hours before testing, except astemizole, which should not be taken within 6 weeks before testing and loratadine which should be withheld for 10 days. A droplet of allergen, histamine or saline will be placed onto the skin and the skin will be pricked with a lancet. Measurements of weal size will be recorded after 10 min (for histamine) and 15 min for allergens and control. A positive reaction will be one that is 2mm greater than the negative control.

6.2 Total Nasal Symptom Scores (TNSS)

Following exposure to allergen, there is an immediate rise in the reported symptoms. The magnitude of these symptoms changes with time, and the recording of the symptoms scored at various time points is referred to as the TNSS. Participants will be asked to record their symptoms on a 4 point scale, with 0 representing no symptoms and 3 representing maximal symptoms:

0 = absent symptoms (no sign/symptom evident)

1 = mild symptoms (sign/symptom clearly present, but minimal awareness; easily tolerated)

2 = moderate symptoms (definite awareness of sign/symptom that is bothersome but tolerable)

3 = severe symptoms (sign/symptom that is hard to tolerate; causes interference with activities of daily living and/or sleeping)

Symptoms will be recorded under the following;

~ Sneezing
~ Itching
~ Rhinorrhoea
~ Congestion

The individual symptoms will be summed to give a total nasal symptom score with a maximum value of 12 (Dreskin *et al*. 2002).

6.3 Asthma Symptom scores

Asthma symptom scores help to determine the severity of responses to inhaled allergens. Participants will be asked to record their symptoms on a 4-point Likert scale, where symptoms will be scored as follows;

0 = none
1 = mild
2 = moderate
3 = severe

Symptoms will be recorded under the following:

Cough
Wheeze
Breathlessness

6.4 Peak Nasal Inspiratory Flow (PnIF)

Peak nasal inspiratory flow is an easy and inexpensive means of measuring nasal obstruction and correlates significantly with severity of symptoms scores. Nasal inspiratory flow will be measured using an In-check™ flow meter (Clement Clarke International Ltd, Harlow, UK). After blowing their nose, participants will inspire forcefully from residual volume to total lung capacity with their mouth closed. All measurements will be made while in the sitting position with a good seal around a purpose built facemask. The median of 3 readings will be recorded.

6.5 Intranasal allergen challenge

In seasonal pollen-related disease, there are variations between subjects in the extent of pollen sensitization. We are using an out-of-season nasal allergen challenge in order to control variability and focus on allergic disease mechanisms, Subjects will be asked to refrain from anti-histamine use for at least 3 days leading up to the Day 1 assessments. The procedure will be performed as previously described (Dreskin *et al*. 2002). Briefly, baseline nasal symptoms scores will be recorded. Saline will be administered 1 squirt up each nostril and the symptom scores will be recorded again after 5 minutes. Allergen will be administered to the nose in ten-fold escalating doses of grass pollen from 10 to 1000bau (given as 0.1ml of concentrations from 100 to 10,000 biologically active units (bau)/ml) every 5 minutes. Symptom scores will be recorded 5 minutes following each dose. The challenge will finish when the participant has a TNSS of 4 or the highest concentration has been given.

6.6 Modified Intranasal allergen challenge

Nasal allergen challenge is a non-invasive method used to study pathophysiological mechanisms in rhinitis. Normally a single high dose of allergen is sprayed into the nose to induce an allergic response. We have modified this test so that we will first find an optimum dose that will induce a certain fixed level of response for each participant. That individual dose will then be used in the study. We believe this will avoid any adverse events and will improve our study since all participants will start the study at the same maximum response. Changes from this will be monitored following intervention.

Subjects will be asked to refrain from anti-histamine use for at least 3 days leading up to the Day 1 assessments. A modified challenge will be performed (Dreskin *et al*. 2002). The concentration of allergen identified in the allergen challenge performed during procedure 1 (screening visit) will be administered at 0.1ml using a pump spray. Nasal symptoms (based on a combination of nasal symptoms including nasal obstruction, itching, sneezing, and rhinorrhoea) will be recorded at 10 minutes, 30 minutes then hourly for 12 hours. PnIF will be measured prior to allergen challenge 10 minutes, 30 minutes, and hourly for 12 hours.

6.7 Spirometry

Spirometry will be used to monitor the severity of asthma, and participant response to treatment. This is performed using a spirometer which is a device which measures the amount of air that you can blow out. A spirometry reading usually shows one of four main patterns:

- Normal
- An obstructive pattern – as in asthma
- A restrictive pattern
- A combined obstructive / restrictive pattern

Spirometry will be performed with a Microlab spirometer (Micro Medical Ltd, Rochester, Kent, UK). The procedure will be performed according to American Thoracic Society specifications [1987]. Measurements will be made of FEV1, FVC, FV6 and peak expiratory flow.

6.8 Laboratory measurements

Cells derived from nasal scrapings, peripheral blood and their secretions will be tested for key markers of allergic inflammation and its regulation. This will enable us to measure efficacy of the intervention.

- Characterisation of cells derived from nasal scrapings
- Epithelial and peripheral blood cell culture and analysis of TSLP, cytokines, chemokines and soluble cytokine receptors and sCD23 following *in vitro* allergenic challenge
- Eotaxin, MIP-1α and RANTES in nasal lavage
- Differential counts of cells derived in nasal lavage/turbinates

6.9 Nasal tissue collection

6.10.1 *Nasal scraping*

Scrapings of nasal epithelium (near the inferior turbinate) will be obtained from all subjects with a Rhino-probe (Arlington Scientific, Springville, UT) according to manufacturer's instructions. Two contiguous scrapings will be taken from the right nostril on Day 1 prior to allergen challenge. Two additional scrapings will be collected from each subject on Day 2 approximately 24 hours after challenge. The first scrape will be from the left nostril the second scrape will be from the right nostril

6.10.2 *Nasal lavage*

This will be performed according to the method described by Grünberg *et al.* (1997). Briefly, a Foley silicone catheter size number 14, is prepared by cutting off the tip of the catheter just distal to the balloon while keeping the balloon intact (about 5 mm from the end) and also cutting off the opposite end of the balloon that does not inflate it, until it will fit into a 10 ml syringe. The tip of the Foley catheter will be located in the right frontal nasal passage (around 1 or 1.5 cm in; not against nasal septum) and the subject will hold the catheter to keep it in position. The balloon will then be inflated until it feels tight but does not hurt. The subject will sit at a desk in the writing position. The technician will instil 10 ml of isotonic saline (0.9%) through the catheter leaving the syringe on the catheter. If fluid comes out of the nostril in which the balloon is blown up, the procedure will be repeated with the balloon inflated more. If fluid comes out of the nostril in which there is no balloon, it will be allowed to drip into the container and the head will be tilted to the side of the balloon catheter. The fluid will be instilled and withdrawn 20 times over a 5 minute period. Then, the balloon will be deflated.

6.10.3 *Nasal scraping processing*

Cells that are not for culture will be placed in phosphate buffered saline and centrifuged at 1000rpm (140g) for 10 minutes. Cells will be resuspended in 4% paraformaldehyde in PBS, pH 7.4 for 10 minutes. The fixed cell suspension is centrifuged (1000rpm, 5 minutes) and resuspended at 1x10^6/ml in sterile PBS;100μl aliquots are then loaded into cytospin funnels and centrifuged onto microscope slides pre-coated with adhesive using a cytospin centrifuge at 600rpm (30g) for 5 minutes.

6.10 Preparation of grass pollen antigens for in vitro challenge

Grass pollen antigens used for the nasal allergen challenge be the same as those used for the nasal allergen challenge, or they may be purchased as grass mixes from Pharmacia Diagnostics (Sweden). The allergen will be at pre-determined at its optimum dose.

6.11 Preparation of mononuclear cells(MNC) and cell culture

Heparinised peripheral blood will be diluted 1:1 with Hank's Balanced Salt Solution (HBSS) and the MNC isolated by density gradient centrifugation using Ficoll-Isopaque (Sigma, Dorset, UK). Following 3 washes with HBSS, the cells will be resuspended in tissue culture medium (TCM) comprising RPMI 1640 medium, 2mmol L-Glutamine, 100ug each of penicillin and streptomycin (all from Sigma) and 5% autologous plasma. Cell concentrations will be adjusted to 2x10^6/ml prior to culture in the presence or absence of pollen allergen at optimum dose. Following culture for 6 days, supernatants will be collected and stored at -80°C to await analysis.

6.13 Preparation and *in vitro* culture of nasal epithelial cells

Specimens of nasal tubinates will be taken and washed immediately in RPMI 1640 medium containing 50 mg/ml gentamycin and 20 mM L-glutamin. The epithelium will be detached mechanically from the lamina propria and digested overnight at 4_C in trypsin solution (0.17%). After disaggregation of the cells, they will be washed in bronchial epithelial growth medium (BEGM; Promocell, Heidleberg, Germany). The cells will be pelleted at 125 g at room temperature for 5 min and resuspended in growth medium. The nasal cells will then seeded at a density of 5x10^3 on uncoated polystyrene culture dishes of 10 cm diameter. Cultures will be maintained at 37°C with 5% CO_2 in air. The cells will be fed with fresh pre-warmed media after 48 h and later every second day. When the cells reach about 75% confluency they will be detached by trypsin digestion (0.5g trypsin and 0.2g EDTA) divided and passaged until use.

6.14 Analysis of culture supernatants

6.14.1 *Grass pollen-specific serum IgE and IgG*

Within two hours of blood collection, coagulated blood will centrifuged at 4°C to stimulate clot retraction and to give a good serum yield. Once collected, the serum will be stored at -80°C until immunoglobulin (Ig) analysis is performed. Grass pollen-specific IgE levels will be identified using either the RIDA-screen system from R-Biopharm (Quadratech, Surrey, UK.) or the Pharmacia ImmunoCap system. Quantification of these levels, in standard units, will be performed using the appropriate instrumentation and software.

6.14.2 *FcεR1 expression on plasmacytoid Dendritic Cells (pDCs)*

Cells will be cultured for 6 days in the presence or absence of grass pollen antigens, washed and stained using established techniques. pDCs will be identified with fluorochrome-labelled antibodies for their CD123+, CD303+ and CD11c− phenotype. Their co-expression of FCεR1 will be evaluated using a compatible fluorochrome-labelled antibody in a 4-colour staining procedure, followed by flow cytometry.

6.14.3 *Cytokines and chemokines*

These will be detected using bead array technology and flow cytometry. The assay is based on a series of spectrally discrete particles that can be used to capture and quantify soluble analytes. The analyte is then measured by flow cytometry. Concentration of the unknowns is calculated through the use of standards. The following cytokines will be measured;IL-1, -4, -5, -6, -8, -10, -12p70, -13, IFN-γ, TNFα, Rantes, Eotaxin, MIP-1a and TGF-β.

6.14.4 *Soluble CD23*

Serum sCD23 will be measured by ELISA according to the manufacturer's instructions.

6.15 Analysis of cultured nasal epithelial cells

6.15.1 Cellular expression of co-stimulatory molecules CD80, CD86, OX40L and CD117

Using 5-colour immunofluorescent staining, cell lineage will be defined using lineage-specific reagents. At the same time their co-expression of CD80, CD86 and OX40L will be analysed using compatible fluorochrome-labelled antibodies and flow cytometry.

6.15.2 Cell surface expression of CD117, IgE, intracellular tryptase and chymase

Cell surface and intracellular expression will be analysed using fluorochrome-labelled antibodies and flow cytometry. Intracellular antigens will be detected by prior cell permeabilisation according to the standard in-house method.

6.15.3 Presence of Soluble Cytokine Receptors (SCR) in culture supernatants

SCR will be determined by bead array technology (Millipore, UK) according to the manufacturer's instructions.

7 Adverse Events and Serious Adverse Events

The local investigator at the study site is responsible for the detection and documentation of events meeting the criteria and definition of adverse events or adverse reactions including reporting to the sponsor.

7.1 "adverse event" (AE) means any untoward medical occurrence in a subject to whom a medicinal product has been administered, including occurrences which are not necessarily caused by or related to that product.

7.1.2 "adverse reaction" (AR) means any untoward and unintended response in a subject to an investigational medicinal product, which is related to any dose administered to that subject.

7.1.3 "serious adverse event" (SAE) or "serious adverse reaction" (SAR) or "unexpected serious adverse reaction" means an adverse event/reaction that fulfils at least one of the following criteria:

A) Results in death

B) Is life-threatening

NOTE: The term 'life-threatening' in the definition of 'serious' refers to an event in which the subject was at risk of death at the time of the event. It does not refer to an event, which hypothetically might have caused death, if it were more severe

C) Requires hospitalisation or prolongation of existing hospitalisation

NOTE: In general, hospitalization signifies that the subject has been detained (usually involving at least an overnight stay) at the hospital or emergency ward for observation and/or treatment that would not have been appropriate at the GP clinic or out-participant setting. Complications that occur during hospitalization are AEs. If

a complication prolongs hospitalization or fulfils any other serious criteria, the event is serious. When in doubt as to whether "hospitalization" occurred or was necessary, the AE should be considered serious. Hospitalization for elective treatment of a pre-existing condition that did not worsen from baseline is not considered an AE.

D) Results in persistent or significant disability or incapacity or

NOTE: The term disability means a substantial disruption of a person's ability to conduct normal life functions. This definition is not intended to include experiences of relatively minor medical significance such as uncomplicated headache, nausea, vomiting, diarrhoea, influenza, and accidental trauma (e.g. sprained ankle) which may interfere or prevent everyday life functions but do not constitute a substantial disruption.

E) Consists of a congenital anomaly or birth defect

F) Medical or scientific judgement should be exercised in deciding whether reporting is appropriate in other situations, such as important medical events that may not be immediately life-threatening or result in death or hospitalization but may jeopardize the subject or may require medical or surgical intervention to prevent one of the other outcomes listed in the above definition. These should also be considered serious. Examples of such events are invasive or malignant cancers, intensive treatment in an emergency department or at home for allergic bronchospasm, blood dyscrasias or convulsions that do not result in hospitalization, or development of drug dependency or drug abuse.

7.1.4 "unexpected adverse reaction" means any adverse reaction, the nature or severity of which is not consistent with the applicable product information (e.g. investigator's brochure for an unapproved investigational product, or summary of product characteristics [Smock] for an authorised product).

7.1.5 Severity: The term "severe" is used to describe the intensity of a specific event. This is not the same as "serious" which is based on participant/event outcome, or action criteria.]

7.1.6 "Suspected Serious Adverse Reaction (SSAR)"
Means an adverse reaction that is classed in nature as serious and which is consistent with the information about the medicinal product

7.1.7 "Suspected Unexpected Serious Adverse Reaction (SUSAR)"
means an adverse reaction that is classed in nature as serious and which is not consistent with the information about the medicinal product in question

7.1.8 Clinical Laboratory Abnormalities and Other Abnormal Assessments
Abnormal laboratory findings (e.g., clinical chemistry, haematology) or other abnormal assessments that are judged by the investigator as **clinically significant** will be recorded as AEs or ARs if they meet the definition's above. Clinically significant abnormal laboratory findings or other abnormal assessments that are detected during the study or are present at baseline and significantly worsen following the start of the study will be reported as AEs or ARs. However, clinically significant abnormal laboratory findings or other abnormal assessments (eg spirometry, PnIF) that are associated with the disease being studied, unless judged by the investigator as more severe than expected for the subject's condition, or that are present or detected at the start of the study and do not worsen, will not be reported as AEs or ARs. The investigator will exercise his or her medical and

scientific judgment in deciding whether an abnormal laboratory finding or other abnormal assessment is clinically significant.

7.1.9. Time Period, and Frequency of Detecting AEs and ARs
From the time a subject consents to participate in the study until he or she has completed the study (including any follow-up period), all SAEs assessed as related to study participation (e.g., protocol-mandated procedures, invasive tests, or change in existing therapy) will be reported promptly to the principle investigator.

7.1.10 Reporting of Serious adverse reactions
Adverse events will be reported according to the National Research Ethics Service requirements for recording, managing and reporting Adverse Events and Adverse Reactions in Clinical Trials. In particular, on receipt of a SUSAR report the CI will immediately notify Ms Sue Steel, Research and Business Services by sending a copy of the SAE Form by email to the Sponsor's representative so that the necessary reporting by the Sponsor can take place within the deadlines.

8 Data Evaluation

The endpoints to be measured are:

- *Primary endpoints*
  - Total nasal symptom score 10 minutes following nasal allergen challenge.

- *Secondary endpoints*
  - Area under the curve for nasal symptoms scores for 12 hours following nasal allergen challenge
  - Area under the curve for peak nasal inspiratory flow for 12 hours following nasal allergen challenge
  - Phenotype of nasal epithelial cells from scrapings
  - Nasal lavage inflammatory mediator profile

Data analysis and decoding will be performed by simultaneous exchange of raw data and code exchanges between the principal investigator and Yakult

All manuscripts arising from this study will be offered to Yakult for pre-publication perusal. Publicity of results will be conducted in accordance with the policies of the Institute of Food Research, University of East Anglia and Norfolk and Norwich University Hospitals NHS trust. Publication of the results will be in the peer-reviewed literature and at national and international meetings. The results may also be featured in press and trade articles. At the end of the study, following data evaluation, all study participants will be invited to attend a small 'get together' when the study findings will be revealed to them.

9. Statistical evaluation

The primary clinical end-point will be considered to be total allergic rhinitis symptom scores. Using total allergic rhinitis symptom score as the response variable and treatment, subject and time as explanatory factors, a repeated measures ANOVA will be used to analyse the data including time as a random effect also treatment group, baseline score and variables used in stratification will be included as fixed effects . Any significant effects for the factors will be investigated further by TukeyHSD (post-hoc analysis) to ensure that the type 1 error rate remains at 5%. Similar ANOVA models will also be used to study the effect of the other measured response variables (e.g. nasal PIFR) against treatment. In addition, a canonical

discriminant analysis may be performed to unravel the complex immunological interactions taking place.

Analysis of time-to-event data will be analysed using the log-rank test to compare between groups and the proportional hazards model will be used if it proves necessary to adjust for baseline measures. The analysis will be stratified by variables used to stratify the randomisation procedure.

10. Monitoring of trial

The trial will be monitored by regular visits from a Yakult UK science representative

11. Roles and responsibilities of all parties

Dr Kamal Ivory is named Principal Investigator at IFR Norwich. A co-ordinator for the trial, she is responsible for all practical arrangements of the trial including randomization, data analysis, management of samples, pickup and storage.

Dr Andrew Wilson, a qualified medical practitioner, is Clinical lead for the trial, responsible for the clinical examination and taking of samples from the volunteers.

Prof. Claudio Nicoletti at IFR Norwich is responsible for final approval of the protocol and contract, and ensuring that all investigators conduct the study as agreed. He will oversee the preliminary and final reports.

Dr Darren Sexton is responsible for performing epithelial cell cultures.

Yakult Honsha will supply part funding towards this project

Dr. Linda Thomas (Yakult) is principal UK contact for Yakult

Dr Jia Zhao (Yakult) is principal Yakult Europe contact, with responsibility for contract negotiations.

(Yakult) is responsible for sample supply.

REFERENCES

American Thoracic Society. Standardisation of spirometry - update. (1987) Am Rev *Respir Dis.* 136:1285-98.

Bjorksten B., Naaber P., Sepp E., Mikelsaar M. (1999) The intestinal microflora in allergic Estonian and Swedish 2-year-old children. *Clin Exp Allergy* 29:342-46.

Dreskin SC, Dale SN, Foster SM, Martin D, Buchmeier A, Nelson HS. (2002) Measurement of changes in mRNA for IL-5 in noninvasive scrapings of nasal epithelium taken from patients undergoing nasal allergen challenge. *J Immunol Methods.* 268(2):189-95.

Ewaschuk JB,, Diaz H., Meddings L., Diederichs B., Dmytrash A., Backer J., Looijer-van LM., Madsen KL. (2008) Secreted bioactive factors from Bifidobacterium infantis enhance epithelial cell barrier function. *Am J Physiology. Gastrointestinal and liver physiology* 295(5):G1025-34.

Forsythe P., Inman MD., Bienenstock J. (2007) Oral Treatment with Live *Lactobacillus reuteri* Inhibits the Allergic Airway Response in Mice. *Am J Resp Cril Care Med.* 175:561-69.

Gaboriau-Routhiau V, Moreau M-C. (1996) Gut Flora Allows Recovery of Oral Tolerance to Ovalbumin in Mice after Transient Breakdown Mediated by Cholera Toxin or Escherichia coli Heat-Labile Enterotoxin. *Pediatric Res*. 39(4):625-29.

Grünberg K., Timmers MC., Smits HH., De Klerk EPA., Dick EC., Spaan WJM., Hiemstra PS., Sterk PJ. (1997) Effect of experimental rhinovirus 16 colds on airway hyper-responsiveness to histamine and interleukin-8 in nasal lavage in asthmatic subjects in vivo. *Clin Exp Allergy* 27:36-45.

Helin T, Haahtela S, Haahtela T. (2002) No effect of oral treatment with an intestinal bacterial strain, *Lactobacillus rhamnosus* (ATCC 53103), on birch-pollen allergy: a placebo-controlled double-blind study. *Allergy*. 57(3):243-6.

Hord NG. (2008) Eukaryotic-Microbiota Crosstalk: Potential Mechanisms for Health Benefits of Prebiotics and Probiotics. *Ann Rev Nutr*.28:215-31.
Isolauri E., Arvola T., Sutas Y., Moilanen E., Salminen S. (2002) Probiotics in the management of atopic eczema. *Clin Exp Allergy*.2000; 30:1604–10.
Ivory K, Chambers SJ, Pin C, Prieto E, Arqués JL, Nicoletti C. (2008) Oral delivery of *Lactobacillus casei* Shirota modifies allergen-induced immune responses in allergic rhinitis. *Clin Exp Allergy*. 38:1282–9.
Kim JH., Ohsawa M. (1995) Oral tolerance to ovalbumin in mice as a model for detecting modulators of the immunologic tolerance to a specific antigen. *Biol pharmaceut bulletin* 18(6):854-58.
Kirjavainen PV., Arvola T., Salminen SJ,, Isolauri E. (2002) Aberrant composition of gut microbiota of allergic infants: a target of bifidobacterial therapy at weaning? *Gut*. 51:51–5.
MacDonald TT., Spencer J. (1994) Ontogeny of the gut-associated lymphoid system in man. *Acta Paediatr Suppl.* 83:3 –5.
Noverr MC., Huffnagle GB. (2004) Does the microbiota regulate immune responses outside the gut? *Trends Microbiol*. 12:562-68.
Noverr MC., Falkowski NR., McDonald RA., McKenzie AN., Huffnagle GB. (2005) Development of allergic airway disease in mice following antibiotic therapy and fungal microbiota increase: role of host genetics, antigen, and interleukin-13. *Infect. Immun*. 73:30-8.
Saavedra JM. (2007) Use of Probiotics in Pediatrics: Rationale, Mechanisms of Action, and Practical Aspects. 22;351-64.
Sudo N, Sawamura S, Tanaka K, Aiba Y, Kubo C, Koga Y. (1997) The requirement of intestinal bacterial flora for the development of an IgE production system fully susceptible to oral tolerance induction. *J Immunol*. 159(4):1739-45
Wang MF, Lin HC, Wang YY, Hsu CH. (2004) Treatment of perennial allergic rhinitis with lactic acid bacteria. *Pediatr Allergy Immunol.* 15(2):152-8.
Yasui H, Kiyoshima J, Hori T. (2004) Reduction of influenza virus titer and protection against influenza virus infection in infant mice fed Lactobacillus casei Shirota. *Clin Diagn Lab Immunol.* 11:675–79.
Xiao JZ, Kondo S, Yanagisawa N, Takahashi N, Odamaki T, Iwabuchi N, Miyaji K, Iwatsuki K, Togashi H, Enomoto K, Enomoto T. (2006) Probiotics in the treatment of Japanese cedar pollinosis: a double-blind placebo-controlled trial. *Clin Exp Allergy*. 36(11):1425-35.
