# Supplementary material for: Oral Delivery of a Probiotic Induced Changes at the Nasal Mucosa of Seasonal Allergic Rhinitis Subjects after Local Allergen Challenge: A Randomised Clinical Trial
Source: PLoS One. 2013 Nov 15;8(11):e78650. doi: 10.1371/journal.pone.0078650 (PMC3829814; doi:10.1371/journal.pone.0078650)
Supplement: File S4 — Clinical trial registration information. (PDF) [file pone.0078650.s005.pdf]

ClinicalTrials.gov
Protocol Registration System

Protocol Registration Receipt
05/26/2010

Seasonal Allergic Rhinitis Study (SAR)

This study is not yet open for participant recruitment.
Verified by University of East Anglia, May 2010

Sponsor:	University of East Anglia
Collaborators:	Institute of Food Research<br>Norfolk and Norwich University Hospitals NHS Foundation Trust<br>Yakult Honsha Co., LTD
Information provided by:	University of East Anglia
ClinicalTrials.gov Identifier:	NCT01123252

Purpose

The study aims to examine the effect of probiotics on the clinical symptoms of allergic rhinitis and to elucidate some of the immunological mechanisms involved.

Condition	Intervention	Phase
Seasonal Affective Rhinitis<br>Asthma<br>Grass Allergy	Dietary Supplement: Lactobacillus casei Shirota (LcS)<br>Dietary Supplement: Placebo	Phase 2

Study Type: Interventional
Study Design: Supportive Care, Parallel Assignment, Double Blind (Subject, Investigator), Randomized, Efficacy Study
Official Title: Evaluation of the Effect of Yoghurt-type Drink on Symptoms of Subjects Suffering Seasonal Allergic Rhinitis (SAR) [Rhinitis 2]

Further study details as provided by University of East Anglia:

Primary Outcome Measure:

- Total nasal symptom score [Time Frame: 10 minutes following nasal allergen challenge] [Designated as safety issue: No]

  Following exposure to allergen, there is an immediate rise in the reported symptoms,the recording of the symptoms scored at various time points is referred to as the TNSS. Participants will be asked to record their symptoms on a 4 point scale, with 0 representing no symptoms and 3 representing maximal symptoms:

  0 = absent symptoms

  1. = mild symptoms
  2. = moderate symptoms
  3. = severe symptoms

  Symptoms will be recorded under the following: Sneezing / Itching / Rhinorrhoea / Congestion

  The individual symptoms will be summed to give a total nasal symptom score.

Secondary Outcome Measures:

- Area under the curve for nasal symptoms scores for 12 hours following nasal allergen challenge [Time Frame: 12 hours] [Designated as safety issue: No]
- • Area under the curve for peak nasal inspiratory flow for 12 hours following nasal allergen challenge [Time Frame: 12 hours] [Designated as safety issue: No]
- Phenotype of nasal epithelial cells from scrapings [Time Frame: 4 months] [Designated as safety issue: No]
- Nasal lavage inflammatory mediator profile [Time Frame: 4 months] [Designated as safety issue: No]

Estimated Enrollment: 60
Study Start Date: September 2010
Estimated Study Completion Date: November 2011
Estimated Primary Completion Date: April 2011

Arms	Assigned Interventions
Active Comparator: Seasonal Affective Rhinitis Group 1<br>Active Comparator Group	Dietary Supplement: Lactobacillus casei Shirota (LcS)<br>Subjects will receive one bottle of Yakult containing the probiotic bacterium Lactobacillus casei Shirota (active drink) once daily for 4 months (16 weeks).
Placebo Comparator: Seasonal Affective Rhinitis Group 2<br>Placebo Group	Dietary Supplement: Placebo<br>Subjects will receive one bottle of a placebo milk drink, once daily for 4 months (16 weeks).

Epidemiological studies have shown that the incidence of atopic diseases (eczema, food allergy, allergic rhinitis and asthma) has been rising over the last few decades. The rate of increase precludes genetic make-up as the

sole cause of the atopic epidemic and implicates environmental factors instead. Currently, allergic rhinitis (hay fever) is one of the world's most common chronic allergic diseases. It affects over 600 million people and often leads to asthma. There are huge costs associated with the condition in terms of both health care and work days lost, with British businesses estimated to lose £324 million this summer alone. For the sufferers, the symptoms severely affect their quality of life. It disturbs their sleep and impairs daytime concentration and performance at work or school. Currently there is no cure for it.

The importance of the gut microbiota in general well-being is evidenced by several experimental observations. It is difficult to achieve oral tolerance in germ-free animals [Sudo et al. 1997] while administration of lipopolysaccharide (a constituent of the outer membrane of gram-negative bacteria) together with food antigens increases the tolerizing effect of feeding [Kim & Ohsawa 1995]. While able to assist in tolerance induction, bacterial products may also break oral tolerance [Gaboriau-Routhiau et al. 1996]. These findings led Wold to suggest in 1998 that an altered normal intestinal colonization pattern in infancy, which fails to induce immunological tolerance, could be responsible for the increase in allergies. Recent studies suggest that the effects of the gut microbiota may not only be related to food antigens, but also to aeroallergens [Noverr et al. 2004, 2005]. Forsythe and his colleagues (2007) managed to attenuate adverse airway responses in a mouse model of allergic asthma through oral administration of probiotics.

The mechanisms by which probiotics exert their effects are unknown at present, but experiments in mice have documented improved gastrointestinal barrier function [Ewaschuk et al. 2008]. Since these experiments have also revealed a strain-dependent heterogeneity in the efficacy of probiotics [N.G. Hord 2008] it is unlikely that improved barrier function alone is responsible for the beneficial effects noted. Given the sheer numbers of microorganisms that inhabit our mucosal surfaces, it is likely that there are normally bidirectional interactions between them and the epithelial, immune, neurologic and endocrine physiological processes initiated by and between them. We hypothesize that a probiotic organism is ingested in sufficient quantities to amplify its particular trait relative to the milieu of other organisms present in relatively lower quantities, temporarily over-riding the diversity present. In that case, the transfer of information between the probiotic organism and cellular components of the gut has particular impact. In order to understand and manipulate this probiotic-mucosa cross-talk towards therapeutic advantage, there is a need to focus on the transfer of information between the microbiota and cellular components of the mucosal immune system.

Few studies have examined the effect of probiotics on allergic rhinitis and the studies that have been performed [Helin et al. 2002; Wang et al. 2004; Xiao et al. 2006] are inconclusive. In our own pilot study we tested the ability of Lactobacillus casei Shirota (LcS) to alter immunological events in seasonal allergic rhinitis (SAR) [Ivory et al. 2008]. The study format was double-blinded and placebo-controlled with ten SAR sufferers in each group. We compared changes in immune status arising through the daily ingestion of a milk drink supplied by Yakult, with or without live LcS, over a period of 5 months. Pre-, peak- and post-grass pollen season blood samples were collected for determination of plasma grass pollen-specific IgG and IgE levels by immunoassay. At the same time, cytokine levels were determined by flow cytometric bead array technology following culture of peripheral blood mononuclear cells for six days in the presence or absence of specific grass pollen antigens. We found that volunteers treated with LcS showed a significant reduction in levels of antigen-induced IL-5, IL-6 and IFN-production compared to volunteers supplemented with placebo. Meanwhile, levels of pollen-specific IgG increased and IgE decreased in the probiotic group. Other changes in cytokine levels were seen but they did not attain statistical significance, most likely due to the small number of volunteers tested. Our work has established for the first time that probiotic supplementation modulates immune responses in allergic rhinitis through down-regulation of both Th1- and Th2-type cytokines and to beneficially alter the balance of pollen-specific IgG and IgE levels in allergic rhinitis subjects. It has also suggested that the impact of probiotics reaches beyond the

intestine.

We would now like to conduct another study to show that the immunological changes arising through probiotic consumption have an impact on the clinical symptoms of hay fever. In addition, we propose to elucidate some of the mechanisms involved that may contribute to the health benefit. As far as we know there have not been any similar studies to date. The ability to demonstrate clinical efficacy of dietary intervention for hay fever treatment has obvious benefits for the relevant sufferers. Economic benefits would arise through the relatively inexpensive 'treatment' that would be self-administered and likely lower absenteeism from work. There is also a need for evidence-based viability for the many health claims made for probiotic consumption that could result in clear guidance to the general public.

Eligibility

Ages Eligible for Study: 16 Years and older
Genders Eligible for Study: Both

Inclusion Criteria:

- Men or women over 16 years of age.
- A history of SAR for a minimum of 2 years before study entry.
- Documentation of sensitivity by positive skin testing (by prick or intradermal methods) or by adequately validated in vitro tests for specific IgE (e.g., RAST, PRIST) to grass pollen within 12 months prior to enrolment. If this is not available, appropriate tests will be performed at screening.
- Able to provide written informed consent

Exclusion Criteria:

- Ingestion of probiotics as part of normal diet
- Significant medical, surgical or psychiatric disease that in the opinion of the participants' attending physician would affect subject safety or influence the study outcome.
- Symptoms of rhinitis at screening indicated by total symptom scores of more than 2 out of 12 (based on a combination of nasal symptoms of blockage, sneezing, rhinorrhoea and itching).
- Current smokers or ex-smokers of <1 year or those who have smoked the equivalent of 20 cigarettes/day for 20 years or more.
- Participants receiving any form of corticosteriod from 1 month prior to the study
- Inadequate washout periods for the following:

Intranasal cromolyn (2 weeks) Intranasal or systemic decongestants (3 days) Intranasal or systemic antihistamines (3 days), except astemizole (6 weeks) or loratadine (10 days).

- Documented evidence of acute or significant chronic sinusitis
- A history of hypersensitivity to the milk or its products
- Pregnant women or those planning a pregnancy. It is important not to include pregnant women in the study due to the possibility of miscarriage following anaphylaxis.
- Lactating women are excluded as those infants breast fed by mothers responding to allergenic challenges can transmit the manifestations of allergic responses to the feeding infant via breast milk

Contacts and Locations

Contacts

Kamal Ivory 00441603 255374 kamal.ivory@bbsrc.ac.uk

Locations

United Kingdom, Norfolk

CRTU University of East Anglia
Norwich, Norfolk, United Kingdom, NR47TJ
Principal Investigator: Andrew M Wilson, MD, MRCP (UK)

Institute of Food Research
Norwich, Norfolk, United Kingdom, NR4 7UA

Norfolk and Norwich University Hospital
Norwich, Norfolk, United Kingdom, NR4 7UY
Contact: Andrew Wilson, MD (MRCP) 01603591257 a.m.wilson@uea.ac.uk
Principal Investigator: Andrew Wilson, MD (MRCP)

University of East Anglia
Norwich, Norfolk, United Kingdom, NR47TJ
Contact: Andrew Wilson, MD MRCP (UK) 01603 591257 a.m.wilson@uea.ac.uk
Principal Investigator: Andrew Wilson, MD MRCP (UK)

Investigators

Principal Investigator: Andrew Wilson University of East Anglia

More Information

Responsible Party: University of East Anglia (Dr Andrew Wilson)
Study ID Numbers: UEA SAR 1
Health Authority: United Kingdom: Research Ethics Committee
