# Supplementary material for: Oral Delivery of a Probiotic Induced Changes at the Nasal Mucosa of Seasonal Allergic Rhinitis Subjects after Local Allergen Challenge: A Randomised Clinical Trial
Source: PLoS One. 2013 Nov 15;8(11):e78650. doi: 10.1371/journal.pone.0078650 (PMC3829814; doi:10.1371/journal.pone.0078650)
Supplement: Protocol S1 — Study protocol. (DOCX) [file pone.0078650.s006.docx]

# Supporting Information S1: STUDY PROTOCOL

# Introduction

The prevalence of allergic disease has been increasing world-wide over the past 25 years placing a substantial burden on patients and the economy[^1^](#_ENREF_1). Seasonal allergic rhinitis (hay fever) is one of the world's most common chronic allergic diseases and approximately one in five adults report symptoms or awareness of this condition in Western Europe[^2^](#_ENREF_2). For the sufferers, the symptoms severely affect their quality of life[^3^](#_ENREF_3). It disturbs their sleep and impairs daytime concentration and performance at work or school. There are considerable health-care costs associated with the condition both in direct treatment costs and in those incurred through treating co-morbidities such as asthma[^4^](#_ENREF_4).

Given the costs and effect on quality of life, over the last two decades there has been increasing interest in evaluating the role of probiotic bacteria in preventing and treating allergic diseases. Their potential arises through the hygiene hypothesis which suggests that protection against atopic, infectious, inflammatory and autoimmune diseases depends upon healthy host-microbe interactions. Epidemiological studies and recent experimental research support this theory in that microbial stimulation of the immune system is found to influence the development of tolerance to innocuous allergens. This makes the gastrointestinal microbiota composition of particular interest, as it provides a major source of immune stimulation and seems to be a prerequisite for the development of oral tolerance. In allergic diseases, there is thought to be a dysregulation in the expression of TH1 and TH2 cytokines. Lactobacilli belonging to the natural intestinal microflora were reported to reduce the incidence severity of allergic manifestations through modulation of TH1/TH2 responses. Although the mechanisms for this are unknown, potential anti-inflammatory actions include direct interaction with epithelial cells to modify their production of inflammatory mediators[^5^](#_ENREF_5). In a study of patients with allergic rhinitis out of the pollen season, 4 weeks’ treatment with *L. casei* reduced the number of CD16/CD56 cells, *L. plantarum* decreased IL-5 and IL-13 and both probiotics reduced birch-pollen specific IgE[^6^](#_ENREF_6). However, the patients were not subjected to allergenic challenge in this study, which makes it impossible to evaluate the impact of these findings on allergic disease. In our earlier study of 20 volunteers with allergic rhinitis receiving *L casei* Shirota (LcS) or placebo for 5 months, we found a significant reduction in levels of antigen-induced IL-5, IL-6 and IFN-gamma production during the pollen season in the probiotic compared with placebo supplemented group. Meanwhile, serum levels of specific IgG increased and IgE decreased in the probiotic group[^7^](#_ENREF_7).

Several trials have investigated the clinical benefit of administering probiotic drinks to patients with seasonal allergic rhinitis[^8^](#_ENREF_8). *Lactobacillus* strains in perennial allergic rhinitis showed small improvement in nasal symptom-medication scores and a tendency for improvement in ocular scores compared to placebo without any significant cellular/immune responses[^9-11^](#_ENREF_9). However, Helin *et al*.^[12](#_ENREF_12)^ could not demonstrate any benefit with *L. rhamnosus* for 5.5 months and the improvement in symptoms scores seen in study by Wang *et al*[^13^](#_ENREF_13) may be explained by the higher scores in the treatment group at baseline. Furthermore, although Aldinucci *et al*.[^14^](#_ENREF_14) showed a clinical benefit, they recruited a small number of patients and all of them had mild disease which did not deteriorate in either group during the pollen season. Despite the potential clinical benefit of probiotic therapy and the reported immunomodulation with this treatment, the clinical data is inconclusive [^15^](#_ENREF_15). We have therefore conducted a double-blinded, placebo-controlled study to test our hypothesis that daily consumption of *Lc*S over a period of four months will result in clinical improvement in an allergen challenge model of allergic rhinitis, with corresponding immunological changes in peripheral blood and nasal epithelial cells.

There are specific difficulties associated with clinical trials in allergic rhinitis. These include variations between subjects in the extent of pollen sensitization due to differences in exposure to pollen even within a given area and duration of the pollen season may be relatively short, with inconsistent pollen count during this period. In order to control such variability we have used a single-dose allergen challenge in the clinic. This also allowed us to study many patients at the same time. Total Nasal Symptom Score (TNSS) was selected as the primary end-point because the nasal symptom questionnaire is a convenient, reliable and valid method for assessing nasal symptom severity. Its principal use was to act as a primary outcome measure by comparing scores before and after treatment. Secondary end-points included peak nasal inspiratory flow (PNIF) as a simple, objective measurement of nasal airflow and laboratory tests for local (at the site of challenge) and systemic (in blood) parameters of immunity.

# The Study

## Objectives

- To assess the clinical efficacy of *Lc*S in modulating the severity of nasal symptom scores following nasal allergen challenge, when consumed daily over a period of 4 months.
- To assess the efficacy of *Lc*S in modulating immunological parameters in nasal lavage, nasal scraping and peripheral blood following nasal allergen challenge, when consumed daily over a period of 4 months.

## Study Design

This was a randomised double-blind placebo-controlled trial comparing the probiotic strain *Lactobacillus casei* Shirota with placebo in patients with seasonal allergic rhinitis. Participants consumed the product once daily for 16 weeks (4 months). The primary outcome measure was the difference in Total Nasal Symptom Score (TNSS) assessed 10 minutes following a nasal allergen challenge. This single centred study was conducted, out of the grass pollen season, between 11 September 2010 and 1 April 2011. The study was conducted in accordance with Good Clinical Practice including Research Ethics Committee (07/MRE05/45) approval and all participants gave written informed consent. The study was registered on an International Trial Register (ClinicalTrials.Gov NCT01123252). First, procedures were undertaken to establish the eligibility of patients for recruitment to the study. Second, pre-intervention baseline measurements were made to document the intensity of local and systemic responses of each patient to a nasal allergen (pollen) challenge. At this time, patients were randomly allocated to either Group A or Group B and given probiotic or placebo drink to consume daily. Finally, after 12 weeks each patient returned for post-intervention follow-up measurements.

## Subjects Selection

Participants were recruited from clinic records, research patient databases and adverts in the lay press. Eligible patients were more than 16 years old, had a history of seasonal allergic rhinitis for a minimum of 2 years prior to the study entry and documented evidence of grass pollen allergy as assessed by positive skin testing or by identification of specific IgE on radioallergosorbent test (RAST) to grass pollen within 12 months prior to enrolment. Patients were excluded if they ingested probiotics as part of normal diet, had symptoms of rhinitis at screening indicated by total symptom scores of more than 2 out of 12 (based on a combination of nasal symptoms of blockage, sneezing, rhinorrhoea and itching), evidence of acute or significant chronic sinusitis or were current smokers or ex-smokers of less than 1 year or smoked the equivalent of 20 cigarettes per day for 20 years or more. Participants were not permitted to receive any form of corticosteroid from 1 month prior to the study or decongestants within 3 days of the study and had an adequate washout of antihistamine therapy. Patients were excluded if they had a history of hypersensitivity to milk products, were pregnant women or those planning a pregnancy, lactating or had significant medical, surgical or psychiatric disease. Patients with and without asthma were included.

## Subject Randomisation

Randomisation was performed by the study statistician using a computer generated code. Patients were randomised in blocks of 4 and 6 on a 1:1 ratio with stratification for the presence/absence of asthma and presence/absence of allergy to tree pollen to receive 65 ml fermented milk drink containing 10^9^ *Lactobacillus casei* Shirota per ml or similar milk drink without *Lactobacillus casei* Shirota.

## Probiotic and placebo drinks

All drinks were manufactured, packed and released by Yakult Europe B.V. Schutsluisweg 1, 1332 EN Almere, Netherlands. Samples were hazard analysis and critical control points (HACCP) certified for the safety of materials, production process and microbial analysis (issued by TNO, Holland). Probiotic and placebo drinks were similar in composition apart from an absence of *L. casei* Shirota in the placebo. They were identical in packaging and appearance. Participants were supplied with probiotic or placebo milk drinks every 2 weeks throughout the 4-month intervention period and the drinks were refrigerated.

## Delivery of drinks and Compliance monitoring

Samples of the milk drinks, active or placebo, were delivered by Yakult Europe B.V to IFR Norwich every 2 weeks. Patients were supplied with probiotic or placebo milk drinks every 2 weeks throughout the 4-month intervention period. Patients were given contact details for both the courier and Dr Kamal Ivory should they need to alter their delivery day or address at any time, and if extra drinks were needed for any reason. Compliance was monitored through recovery of unused drinks at each delivery period.

Study Visits and Measurements

Subjects attended for a screening visit to ensure eligibility, obtain demographic details and medical history and undergo a clinical examination. Skin prick testing was undertaken as previously described to 5 common inhaled allergens with a positive reaction taken as a wheal size 2mm more than negative control. Blood was obtained for routine haematology (haemoglobin, platelet count, white blood count and differential while blood count), renal function (sodium, potassium, urea and creatinine) and liver function (albumin, bilirubin, alkaline phosphatase, alanine transaminase). Total nasal symptoms score (TNSS), was determined as the sum of individual scores for “sneezing”, “itching”, “nasal blockage” “runny nose” on a 4 point Likert scale with 0 representing no symptoms and 3 representing maximum symptoms. The mean of three peak nasal inspiratory flow (PNIF) measures obtained using an In-check™ flow meter (Clement Clarke International Ltd, Harlow, UK) were documented. Subjects with asthma underwent spirometry using a Microlab spirometer (Micro Medical Ltd, Rochester, Kent, UK) as per American Thoracic Society/European Respiratory Society guidelines[^16^](#_ENREF_16) and recorded their total asthma symptoms as the sum for individual scores for “cough”, “wheeze”, “breathlessness” using the same Likert scale. Subjects then underwent an incremental nasal allergen challenge as described by Dreskin *et al*. using timothy grass (ALK-Abello Ltd, Hungerford, Berkshire, UK). Following administration of control, the allergen extract was given, in 0.5 log increasing concentrations from 100 to 10,000 biological active units (BAU)/mL. Subjects were asked to inhale through their mouth to total lung capacity then one squirt (0.1mL) of the starting concentration was sprayed into each nostril from a Valois VP6 metered-dose pump spray (Volis UK Ltd, letchley, Milton Keynes, UK). After 5 min, nasal symptoms scores were obtained. The procedure was repeated with increasing allergen concentrations until a TNSS score of 4 was obtained or the highest concentration was given

At least 2 weeks following the screening visit, subjects returned for baseline assessments which consisted of measurements and sample acquisition before, 6- 8 hours (in a subgroup) and 24 hours following a single-dose nasal allergen challenge. Following clinical examination, baseline TNSS and PNIF were obtained in all patients and ASS and spirometry in patients with asthma. Blood was taken for laboratory analyses***.*** Nasal lavage was performed according to the method described by Grünberg *et al.* (1997). A 14 French female foley urinary catheter (Telefex medical, High Wycombe, United Kingdom) was inserted into the right nasal vestibule so that the balloon was at the level of the nares. The subject held the catheter to keep it in position while sitting upright with the neck flexed forward and laterally to the left. The balloon was then inflated to block the nares and 10 mL of isotonic saline (0.9%) was instilled through the catheter. The pressure of the balloon was increased if saline leaked round the balloon and reduced if uncomfortable. The saline was instilled and withdrawn 20 times over a 5 minute period, and the balloon deflated. Two nasal mucosal scrapes were obtained using a Rhino-probe curette (Arlington Scientific, Inc., Springville, Utah, USA) according to the manufacturer’s instructions. In brief, the curette was placed onto the surface of the mid-inferior portion of the inferior turbinate under direct visual inspection in order to avoid contact with the anterior aspect of the inferior turbinate. The cupped tip of the curette was pressed gently on the mucosal surface and moved outwards approximately 2-3mm. This motion was repeated 2-3 times.

A single-dose nasal allergen challenge was undertaken using the administration procedure described for the incremental nasal allergen challenge. The concentration of allergen which was closest to that causing a TNSS of 4 during the incremental nasal allergen challenge, with a minimum of 333 BAU/mL, was administered at 0.1ml using a pump spray. Nasal symptoms (described above) and PNIF (measured in duplicate) were recorded at 5, 10 and 30 minutes then hourly for 12 hours following the challenge. Patients with asthma recorded asthma symptoms (described above), forced expiratory volume in 1 second (FEV1) and peak expiratory flow (PEF) at 5, 10, 30 and 60 minutes following the nasal allergen challenge. Nasal lavage was obtained from the right nostril 30 minutes following the challenge in all patients and between 6 and 8 hours following the challenge in a subgroup. All patients returned to the laboratory 24 hours following the single-dose nasal allergen challenge for nasal symptom scoring, PNIF, blood sampling, nasal lavage and nasal mucosal scraping, plus asthma symptom scoring and spirometry for asthmatic patients.

The procedures were repeated identically after the patients had received 16 weeks of study intervention.

## Laboratory Analyses

## Sample Collection

All samples were collected at the Norfolk and Norwich University Trust Hospital Clinical Research Centre Laboratory.

## Nasal Lavage

As nasal lavages were collected their volumes were noted for each time point and accounted for during analyses. Data for soluble factors present were acquired by flow cytometry using a multiplex bead array technology or ELISA .

## Nasal Scrapes

Nasal scrapes were cultured for 48 hours in Airway Epithelial Cell Growth Medium (Promocell, Heidelberg, Germany). Culture supernatants were removed and stored at -80^O^C for soluble factor tests. Soluble factors and cytokine receptors were analysed using Milliplex multi-analyte profiling technology for Luminex instrumentation (Millipore UK Ltd, Watford, UK). After removal of supernatants, adherent and non-adherent cells were taken from culture, disaggregated and permeabilized when appropriate before stainng with fluorochrome-labelled cell surface and intracellular antibodies. Data were acquired by flow cytometry on a Beckman Coulter FC500-MPL cytometer.

## Peripheral Blood Mononuclear Cells (PBMNC)

Mononuclear cells were derived from heparinised peripheral blood by density gradient centrifugation. These were cultured at 37^O^C, 5% CO_2_ in air for 6 days in the absence or presence of Timothy grass pollen (*phleum pratense*, Phadia AB, Uppsala, Sweden). After this period culture supernatants were removed, aliquoted and frozen at -80^O^C for detection of soluble molecules. Cytokines were analysed as by multiplex cytometric bead array. TGF-β needed to be tested as a single-plex. All kits were purchased from BD Biosciences (Oxford, UK) and data acquired by flow cytometry (Beckman Coulter FC500 MPL instrument, UK). Soluble CD23 (sCD23; Life Technologies Ltd, Paisley, UK) was quantified by ELISA. Cells were washed and stained for their expression of cell surface molecules. Multi-parametric flow cytometric data were collected on a Beckman Coulter FC500 MPL cytometer.

## Serum

Serum was separated from blood and frozen shortly after its collection at the clinic laboratory. Frozen samples were sent to the Laboratory of Allergy within the [Academic Medical Center](http://maps.google.co.uk/maps/place?cid=508901693264556403&q=academic+medical+centre+amsterdam&hl=en&gl=uk&ved=0CBoQ-gswAA&sa=X&ei=EGBvT9ijFMjc4QTKh830Aw)

 in Amsterdam where pollen-specific IgG, IgG4 and IgE were quantified in all subjects using the Pharmacia ImmunoCAP 100 system.

## Statistical analysis

The primary clinical end-point was the TNSS at 10 minutes following the single-dose nasal allergen challenge. A sample size of 46 participants was calculated to provide 90% power to detect a treatment difference at a two sided 5% significance level, if the true difference between the treatments is a TNSS of 1.5 following allergen challenge assuming a standard deviation of 1.5 (Dreskin et al 2002). Secondary endpoints were PNIF at 10 minutes, the area under the curve for 12 hours following nasal allergen challenge (AUC12) for nasal symptoms scores and PNIF, phenotype of cells from nasal epithelial scrapings and peripheral blood or their secretions, as well as nasal lavage inflammatory mediator profile

Using total allergic rhinitis symptom score as the response variable and treatment, subject and time as explanatory factors, a repeated measures ANOVA was used to analyse the data including time as a random effect, also treatment group, baseline score and variables used in stratification were included as fixed effects. Similar ANOVA models were used to study the effect of the other measured response variables against treatment. No adjustments were made for baseline when no differences were noted between the four stratified groups in the study. The last number carried forward procedure was applied where there were missing data or patients failed to complete the 12 hour collection period.

Laboratory data were analysed at each time point by comparing the post- minus pre-intervention values between the two treatment groups. If the assumptions were met for all time points then a linear regression model was applied, otherwise a Van Elteran test was used to account for the non-normal distribution and the stratifying factors. In addition, Wilcoxon Rank (unpaired, 2-tailed, without correction for multiple tests^18^) tests were used for further investigation of immunological interactions taking place.
